# **β**-Catenin stabilization protects against alveolar hemorrhage through amphiregulin- and BATF-mediated Tregs

**DOI:** 10.1172/jci.insight.201552

**Published:** 2026-01-27

**Authors:** Fiona Mason, Hui Xiong, Ali Mobeen, Md Saddam Hossain, Sara Mahmudlu, Rosanne Trevail, Mikyal Mobeen, Li Chen, Sunny Lee, Tuncay Delibasi, Jyoti Misra Sen, Mobin Karimi

**Affiliations:** 1 Department of Microbiology and Immunology, SUNY Upstate Medical University, Syracuse, New York, USA.; 2 School of Medical Imaging, Nanchang Medical College, Nanchang, Jiangxi, China.; 3 Department of Pathology, and; 4 Division of Endocrinology, Diabetes and Metabolism, Department of Medicine, SUNY Upstate Medical University, Syracuse, NY, USA.; 5 National Institute on Aging, NIH, Baltimore, Maryland, USA.; 6 Center on Aging and Immune Remodeling, Immunology Program, Department of Medicine, Johns Hopkins University, Baltimore, Maryland, USA.

**Keywords:** Autoimmunity, Immunology, Tregs

## Abstract

Alveolar hemorrhage (AH) is a life-threatening condition with high mortality, yet the immunological mechanisms governing disease severity remain poorly defined. Here, we demonstrate a protective role for T cell–intrinsic β-catenin stabilization in AH using a transgenic mouse model (CAT-Tg) in which β-catenin is stabilized under the Lck promoter. We found β-catenin stabilization induced a distinct T cell phenotype marked by expansion of central effector memory cells (CD44^+^CD122^+^Eomes^+^T-bet^+^) and suppression of proinflammatory signaling, including reduced phosphorylation of STAT1, STAT3, and JAK1. Pristane-induced AH was attenuated in CAT-Tg mice, which exhibited reduced lung injury, decreased proteinuria, and diminished pulmonary proinflammatory cytokine production compared with WT controls. Protection was associated with a marked expansion of FOXP3^+^ Tregs. Mechanistically, β-catenin stabilization enhanced lung expression of amphiregulin and BATF, mediators of Treg stability and tissue repair. Adoptive transfer of CAT-Tg–derived Tregs into WT mice conferred superior protection against AH, reducing lung inflammation and proteinuria. Transcriptomic analyses revealed enrichment of tissue repair and immune homeostasis pathways, including PI3K-Akt, angiogenesis, and STAT5 signaling. Collectively, these findings identify β-catenin as a regulator of a protective amphiregulin/BATF/Treg axis, highlighting an immunomodulatory pathway with therapeutic potential for AH and inflammatory lung disease.

## Abstract 2

Our research identifies a new immune pathway that halts severe lung injury and promotes healing, offering potential therapeutic targets for fatal alveolar hemorrhage.

## Graphical abstract

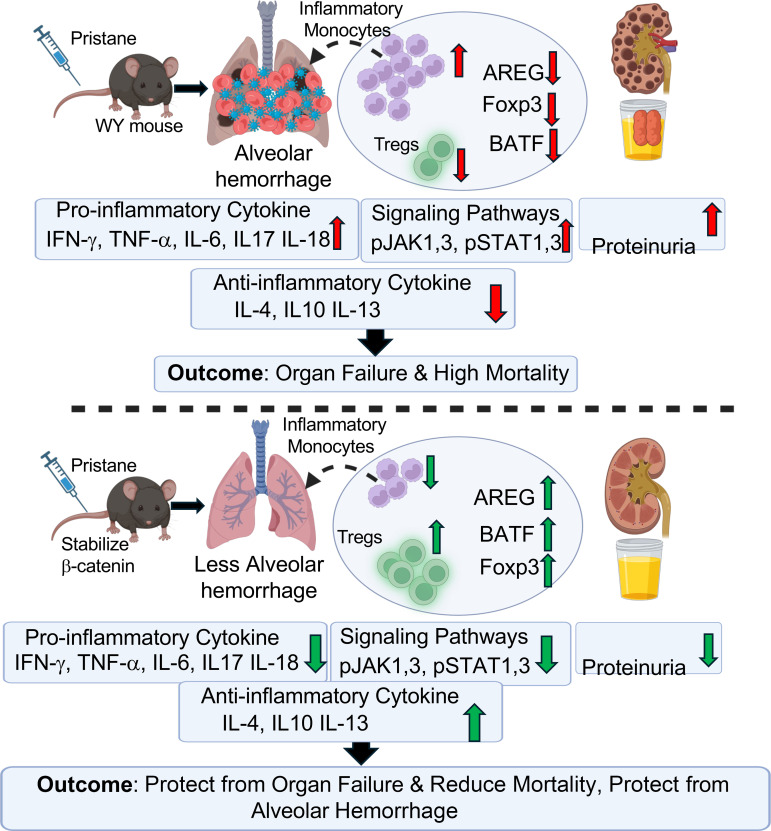

## Introduction

Alveolar hemorrhage (AH) is a life-threatening syndrome characterized by bleeding into the alveolar airspaces resulting from immune-mediated injury to the pulmonary microvasculature. It most commonly occurs in autoimmune diseases such as Goodpasture syndrome, systemic lupus erythematosus, and antiphospholipid syndrome, where immune-mediated capillary damage precipitates pulmonary bleeding (1–3). Current treatment strategies rely on high-dose corticosteroids combined with systemic immunosuppression, including cyclophosphamide, rituximab, and antifibrinolytic agents to stabilize clot formation (3, 4). Despite aggressive therapy, mortality remains unacceptably high, underscoring a critical gap in understanding the immune mechanisms that drive tissue injury versus protection in AH (5–8).

Defining the immune pathways that govern tissue injury and repair in AH is the central objective of this study. Amphiregulin (AREG) and the transcription factor basic leucine zipper ATF-like transcription factor (BATF) is a critical upstream regulator of AREG, as canonical β-catenin signaling directly controls its transcription (9, 10). More broadly, β-catenin signaling is essential for T cell development and tissue homeostasis (11). Upon activation, β-catenin translocates to the nucleus and associates with T cell factor/lymphoid enhancer factor to induce target gene expression, including AREG (9, 12). In addition, β-catenin–dependent transcriptional programs support proliferation, migration, and stem cell maintenance during tissue repair (11, 13). In parallel, BATF, a component of the AP-1 transcription factor complex, is essential for the differentiation and function of multiple immune cell subsets, including T cells and innate lymphoid cells (14, 15). FOXP3^+^ Tregs represent another critical axis of immune tolerance and tissue repair, and AREG has been shown to enhance Treg suppressive function (16, 17). Despite these established roles, the upstream molecular pathways that integrate β-catenin signaling with BATF and AREG regulation in Tregs remain undefined.

Here, we show that β-catenin regulates FOXP3 expression and enhances BATF- and AREG-dependent Treg function, thereby conferring protection against AH. Using transgenic mice with stabilized β-catenin under the lymphocyte-specific protein tyrosine kinase (Lck) promoter (CAT-Tg) (18–20), we found that CAT-Tg CD8^+^ T cells exhibit expanded central memory (CM) and effector memory (EM) subsets; increased expression of CD44, CD122, and T-box transcription factors Eomesodermin (Eomes) and T-bet; and reduced phosphorylation of STAT1, STAT3, and JAK1. Consistent with these immunological changes, CAT-Tg mice were protected from pristane-induced AH, displaying attenuated lung pathology, reduced proteinuria, diminished proinflammatory cytokine production, and enhanced antiinflammatory responses compared with WT controls. Importantly, this protection was associated with increased frequencies of FOXP3^+^ Tregs expressing high levels of AREG and BATF.

Adoptive transfer of CAT-Tg Tregs into WT recipients with pristane-induced AH conferred robust protection, reducing lung inflammation and proinflammatory cytokine production while enhancing antiinflammatory cytokine expression. Consistently, pharmacological activation of β-catenin in WT mice protected against AH, suppressing inflammatory cytokines and expanding Treg populations. Unbiased RNA-Seq further revealed that β-catenin stabilization reprogrammed gene expression toward tissue repair and immune homeostasis. Collectively, these findings identified a β-catenin/AREG/BATF/Treg axis that protects against AH. This work defines β-catenin as a therapeutic target for AH and other cytokine storm–mediated inflammatory lung diseases.

## Results

### β-Catenin stabilization alters the CD8^+^ T cell phenotype.

**Figure 1:**
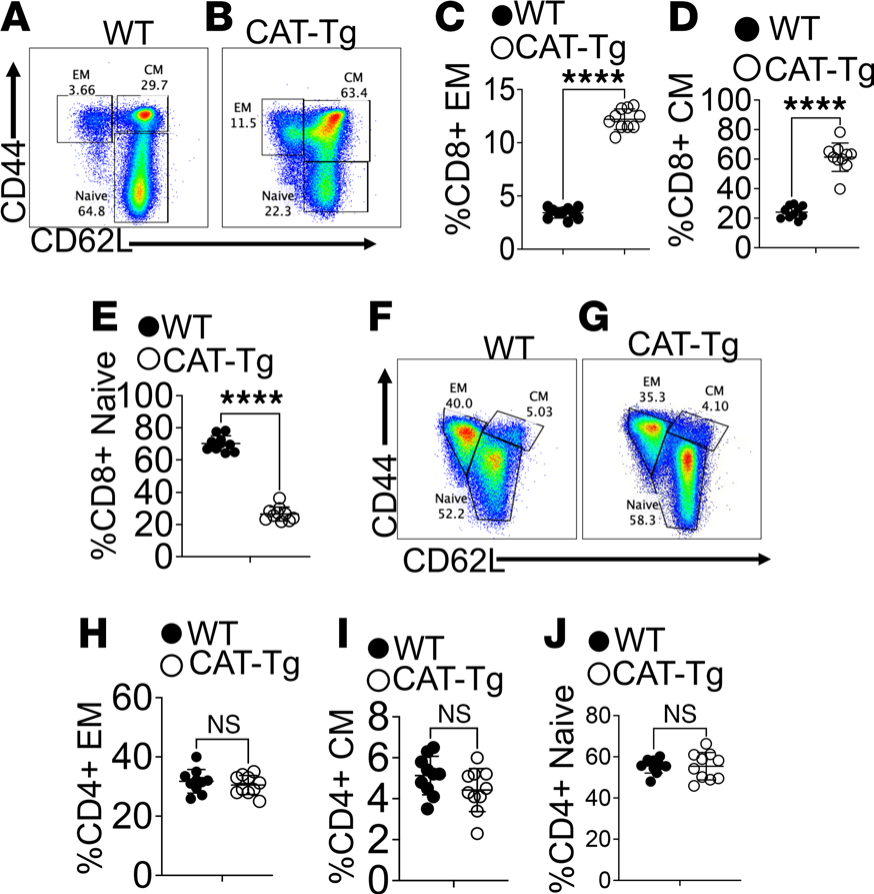
β-Catenin stabilization alters the CD8^+^ T cell memory phenotype. Freshly isolated splenocytes were gated on CD3^+^ T cells and subdivided into CD4^+^ and CD8^+^ populations. Within CD8^+^ T cells, CD44 and CD62L expression was analyzed by flow cytometry (**A** and **B**). CD44^+^CD62L^–^ cells were defined as effector memory (EM), CD44^+^CD62L^+^ as central memory (CM), and CD44^–^CD62L^+^ as naive CD8^+^ T cells. Representative flow plots from WT and CAT-Tg mice are shown, with quantification of EM, CM, and naive CD8^+^ T cell frequencies (**C**–**E**). CD4^+^ T cells were analyzed using the same gating strategy, with representative plots and quantification of EM, CM, and naive subsets shown (**F**–**J**). Data are presented as mean ± SEM (*n* = 10 mice per group; indicated in panels). Each experiment was repeated 6 times. Statistical significance was determined using the appropriate test; ****P < 0.0001.

β-Catenin is required for both αβ and γδ T cell development (21–23). To determine whether stabilization of β-catenin under the Lck promoter (Cat-Tg mice) (24, 25), alters αβ T cell differentiation, we analyzed T cell subsets in WT and CAT-Tg mice, both in the C57BL/6 (B6) background. Because αβ T cell phenotype reflects differentiation status, effector function, and persistence relevant to therapeutic efficacy (26–29), freshly isolated splenic CD3^+^ T cells were analyzed by flow cytometry. Cells were gated on total T cells and subdivided into CD4^+^ and CD8^+^ populations, which were further classified as naive, CM, or EM subsets based on CD44 and CD62L expression (30, 31). CD62L mediates homing to secondary lymphoid tissues, whereas CD44 is associated with activation and migration to peripheral or inflamed sites (32–35). CAT-Tg mice exhibited a significantly increased proportion of CM and EM CD8^+^ T cells compared with WT controls (Figure 1, A–E). In contrast, the distribution of naive, CM, and EM subsets within the CD4^+^ T cell compartment was unchanged between CAT-Tg and WT mice (Figure 1, F–J). These findings indicate that β-catenin stabilization preferentially drives memory differentiation in CD8^+^ T cells, a subset critical for long-term antitumor immunity.

### CD8^+^ T cells from CAT-Tg mice display increased activation markers with attenuated signaling.

**Figure 2:**
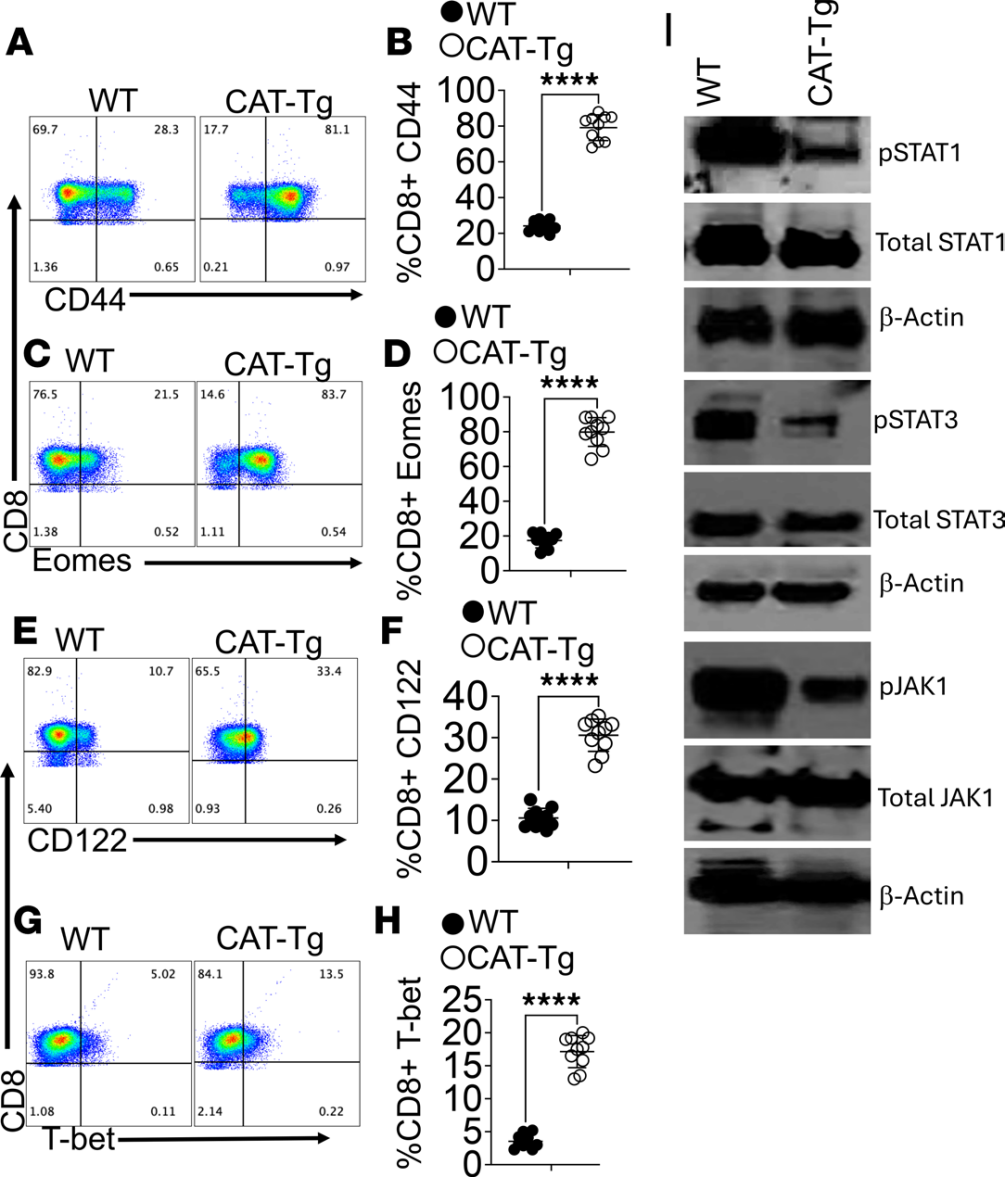
CAT-Tg CD8^+^ T cells display increased activation/memory markers with reduced JAK/STAT signaling. Freshly isolated splenocytes from WT and CAT-Tg mice were gated on CD3^+^CD8^+^ T cells. CD44 expression is shown by representative flow plots (**A**) with quantification (**B**). CD122 expression is shown by representative flow plots (**E**) with quantification (**F**). Intracellular staining for Eomes and T-bet is shown by representative flow plots (**C** and **G**) with quantification in WT and CAT-Tg mice (**D** and **H**) (*n* = 10 per group; indicated in panels). Each experiment was repeated 6 times. For signaling analyses, CD3^+^ T cells were MACS-purified, stimulated with anti-CD3/anti-CD28 for 3 minutes, lysed, and analyzed by immunoblotting for pSTAT1/STAT1, pSTAT3/STAT3, and JAK1, with β-actin as a loading control (**I**). Each experiment was repeated 3 times. Data are presented as mean ± SEM. Statistical significance was determined by 2-tailed Student’s *t* test; ****P < 0.0001.

We previously showed that T cells with attenuated T cell receptor signaling can exhibit elevated activation markers without inducing alloimmunity (25, 30, 36–38). Consistent with this, flow cytometric analysis revealed significantly increased CD44 expression on CD3^+^CD8^+^ T cells from CAT-Tg mice compared with WT controls, as demonstrated by representative plots and quantitative analyses (Figure 2, A and B). We next examined transcriptional regulators of CD8^+^ T cell memory differentiation. T-bet and Eomes cooperatively promote memory formation by inducing CD122, a critical component of IL-2 and IL-15 signaling (39, 40). In line with enhanced memory potential, CD8^+^ T cells from CAT-Tg mice expressed significantly higher levels of T-bet, Eomes, and CD122 than WT CD8^+^ T cells (Figure 2, C–H).

Finally, we examined whether β-catenin stabilization alters proximal T cell signaling. STAT1, STAT3, and JAK1 are central components of the JAK/STAT pathway that regulate T cell growth, differentiation, and immune responses (41, 42). After CD3/CD28 stimulation, CAT-Tg T cells exhibited reduced phosphorylation of STAT1, STAT3, and JAK1 compared with WT controls (Figure 2I). These findings indicate that β-catenin stabilization promotes a highly activated, memory-prone CD8^+^ T cell phenotype while concurrently attenuating JAK/STAT signaling. Previous research showed β-catenin stabilization markedly reduced severe inflammatory autoimmunity, including AH, bronchiectasis, and related autoimmune manifestations (42).

### β-Catenin stabilization protects mice from AH.

**Figure 3:**
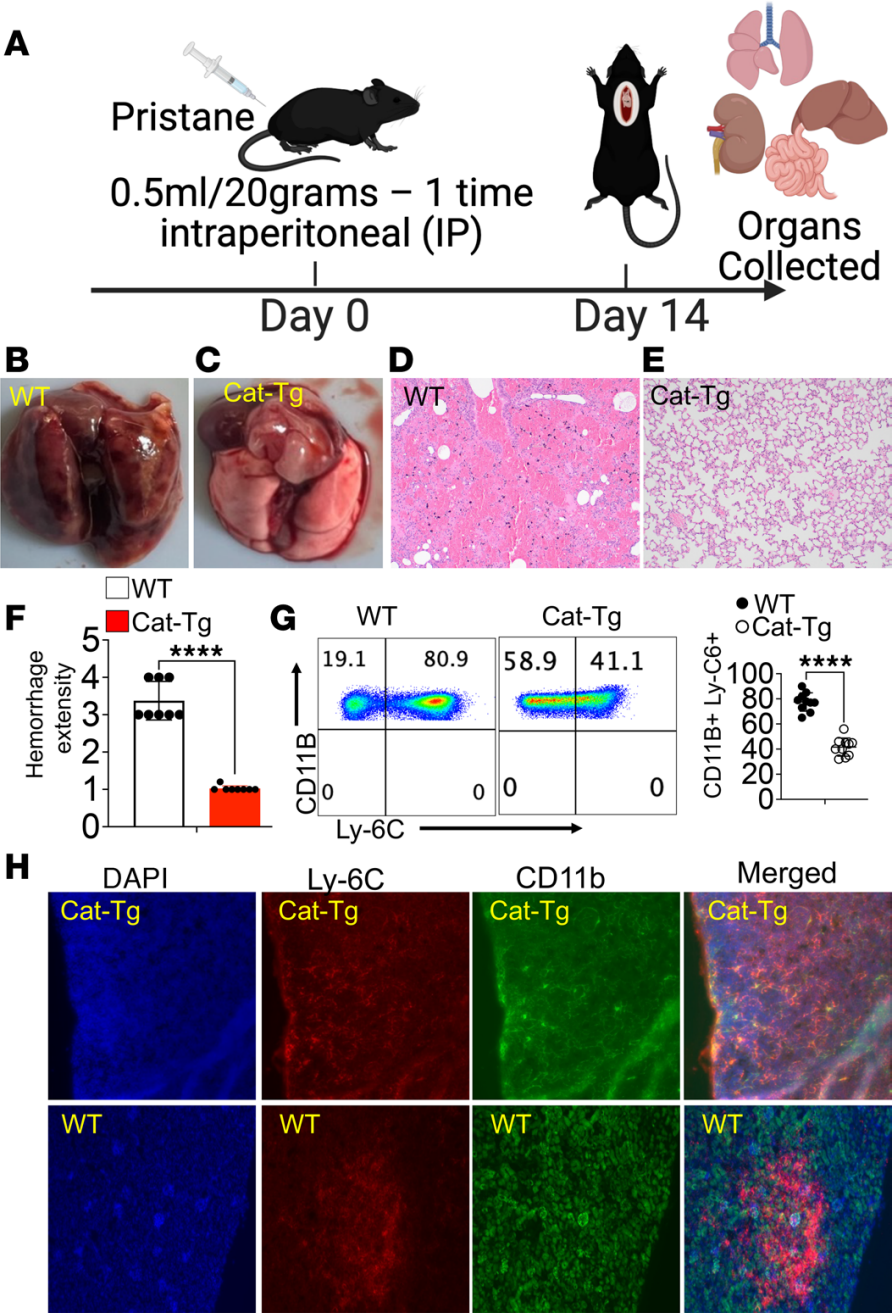
β-Catenin stabilization protects mice from pristane-induced alveolar hemorrhage. (**A**) Experimental schematic. WT and CAT-Tg mice were injected with pristane to induce alveolar hemorrhage (AH) and euthanized on day 14 for tissue collection. (**B**–**D**) Representative gross images of lungs from WT and CAT-Tg mice. (**E**) Representative H&E-stained lung sections. (**F**) Quantification of blinded histopathological scoring. (**G**) Representative flow cytometry plots and quantification of lung-infiltrating inflammatory monocytes, gated as CD11b^+^Ly6C^+^. (**H**) Representative immunofluorescence images of lungs at day 14 after pristane showing DAPI (blue), Ly6C (red), and CD11b (green), with merged images. Data are presented as mean ± SEM (*n* = 10 mice per group). Each experiment was repeated 3 times. Statistical significance was determined by 2-tailed Student’s *t* test; ****P < 0.0001.

To determine whether β-catenin stabilization exacerbates or ameliorates AH, 6–8-week-old WT and CAT-Tg mice were analyzed. Baseline urine and serum samples were collected prior to i.p. injection of pristane (0.5 mL per 20 g body weight) to induce AH (26, 43, 44). Mice were monitored daily for weight loss and clinical signs of disease; no significant weight loss was observed over the 14-day experimental period. On day 14, mice were euthanized and lungs were harvested (Figure 3A). Gross examination revealed marked protection from AH in CAT-Tg mice compared with WT controls (Figure 3, B–E). Histological analysis of lung sections stained with H&E confirmed reduced AH in CAT-Tg mice. All lung sections were independently evaluated by a blinded pathologist, and double-blind pathological scoring validated the protective effect of β-catenin stabilization (Figure 3F). Additional organs, including the kidney, liver, and spleen, showed no significant differences between WT and CAT-Tg mice at the 14-day time point (Supplemental Figure 1, A–L; supplemental material available online with this article; https://doi.org/10.1172/jci.insight.201552DS1). In contrast, substantial pathological differences were observed in these organs at 3 months (data not shown).

To characterize immune cell infiltration during AH (45), pristane-induced AH was established in WT and CAT-Tg mice. Lungs were harvested 14 days after pristane injection; one cohort was processed for flow cytometric analysis, and a second cohort was prepared for IHC. Because inflammatory monocytes are known contributors to lung injury in AH, lung leukocytes were analyzed for CD11b and Ly6C expression (46–48). Lungs from WT mice contained a high frequency (80%–90%) of CD11b^+^Ly6C^+^hi inflammatory monocytes. In contrast, CD11b^+^ monocytes from CAT-Tg mice expressed significantly lower levels of Ly6C, indicating reduced inflammatory infiltration (Figure 3G). Consistent with these findings, IHC analysis confirmed the presence of CD11b^+^Ly6C^+^ infiltrates within lung tissue (Figure 3H), with representative images showing DAPI (blue), Ly6C (red), and CD11b (green). Together, these data establish pristane-induced AH as a highly inflammatory model and demonstrate that β-catenin stabilization markedly attenuates inflammatory monocyte accumulation in the lung. Having established a protective effect of β-catenin stabilization, we next investigated the immunological mechanisms underlying this protection.

### β-Catenin stabilization reduces proteinuria and suppresses proinflammatory cytokines during AH.

**Figure 4:**
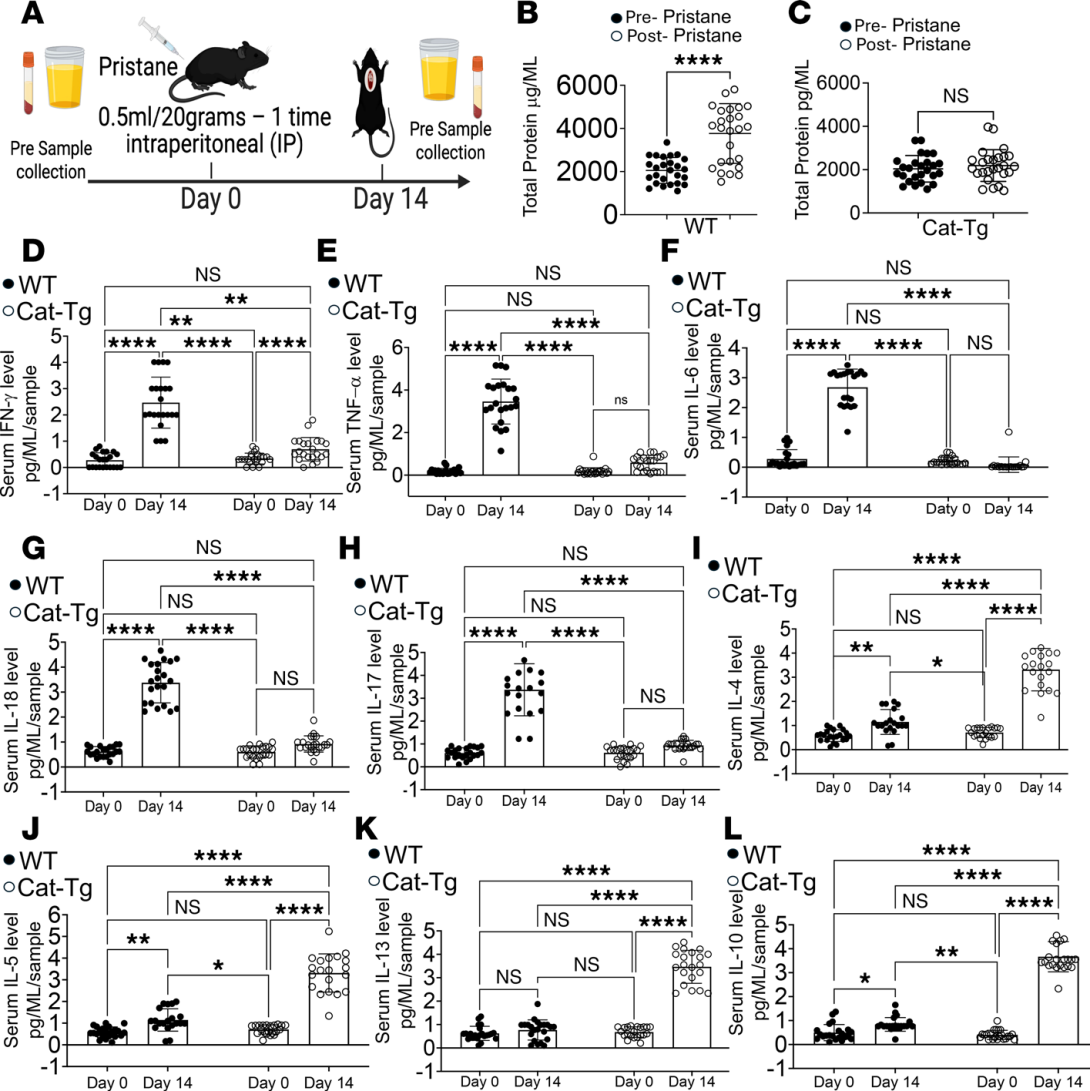
β-Catenin stabilization reduces proteinuria, suppresses proinflammatory cytokines, and enhances antiinflammatory cytokines during alveolar hemorrhage. (**A**) Experimental schematic. WT and CAT-Tg mice were injected with pristane to induce alveolar hemorrhage (AH). Urine and blood were collected at baseline (pre-pristane) and on day 14, followed by euthanasia. (**B** and **C**) Urinary protein was quantified by BCA assay, with comparisons between pre- and post-pristane samples in each group. (**D**–**L**) Serum cytokines were quantified by multiplex bead-based assay, including IFN-γ, TNF-α, IL-6, IL-18, IL-17, IL-4, IL-5, IL-13, and IL-10, and compared between pre- and post-pristane conditions. Data are presented as mean ± SEM; sample sizes (*n* = 15–25 mice per group) are indicated in panels. Each experiment was repeated 3 times. Statistical significance was determined using 2-tailed Student’s *t* test, 1-way ANOVA, or 2-way ANOVA as appropriate; **P < 0.01, ***P < 0.001, ****P < 0.0001.

Proteinuria, a hallmark of pulmonary-renal syndrome, frequently accompanies AH in systemic autoimmune disease and serves as an early indicator of kidney involvement (49). To assess renal injury, proteinuria was measured by ELISA in WT and CAT-Tg mice before and after pristane-induced AH. WT mice exhibited a significant increase in proteinuria by day 14 after pristane injection compared with baseline levels. In contrast, CAT-Tg mice showed no significant change in proteinuria after pristane treatment, indicating that β-catenin stabilization protects against AH-associated renal injury (Figure 4, A–C). Given that pristane-induced AH is characterized by robust inflammatory responses (Figure 3), we next evaluated circulating proinflammatory cytokines. IFN-γ and TNF-α are key mediators of AH pathogenesis, acting synergistically to promote tissue factor expression and procoagulant activity (50). WT mice displayed significant increases in both IFN-γ and TNF-α at day 14 after pristane injection, whereas CAT-Tg mice exhibited no significant changes in these cytokines relative to baseline (Figure 4, D and E). Because IL-6 and IL-18 are key mediators of lung injury and hemorrhage (51, 52), we next assessed their serum levels. IL-6 promotes neutrophil recruitment, a hallmark of hemorrhagic inflammation, whereas IL-18 amplifies inflammasome-driven cascades that increase vascular permeability and alveolar bleeding. After AH induction, CAT-Tg mice exhibited significantly lower levels of both IL-6 and IL-18 compared with WT controls (Figure 4, F–H). IL-17 has also been implicated in lung injury, edema, and AH (53–55); consistent with a protective phenotype, CAT-Tg mice displayed markedly reduced IL-17 induction relative to WT mice (Figure 4H). IL-5 showed increased production in CAT-Tg mice at day 14 after pristane-induced AH (Figure 4J). We next examined whether β-catenin stabilization enhances antiinflammatory cytokine production. Serum from CAT-Tg mice contained significantly higher levels of IL-4, IL-13, and IL-10 compared with WT mice (Figure 4, K–L). In contrast, IL-12 and IL-9 levels were not significantly different between groups (Supplemental Figure 2), indicating selective modulation of antiinflammatory pathways (56, 57). These data demonstrate that β-catenin stabilization protects against AH by concurrently suppressing proinflammatory cytokines (IFN-γ, TNF-α, IL-6, IL-18, and IL-17) and enhancing antiinflammatory cytokines (IL-4, IL-13, and IL-10). Having established both structural and cytokine-level protection, we next investigated the mechanistic basis by which β-catenin attenuates AH severity.

### β-Catenin stabilization enhances AREG and BATF expression in Tregs.

**Figure 5:**
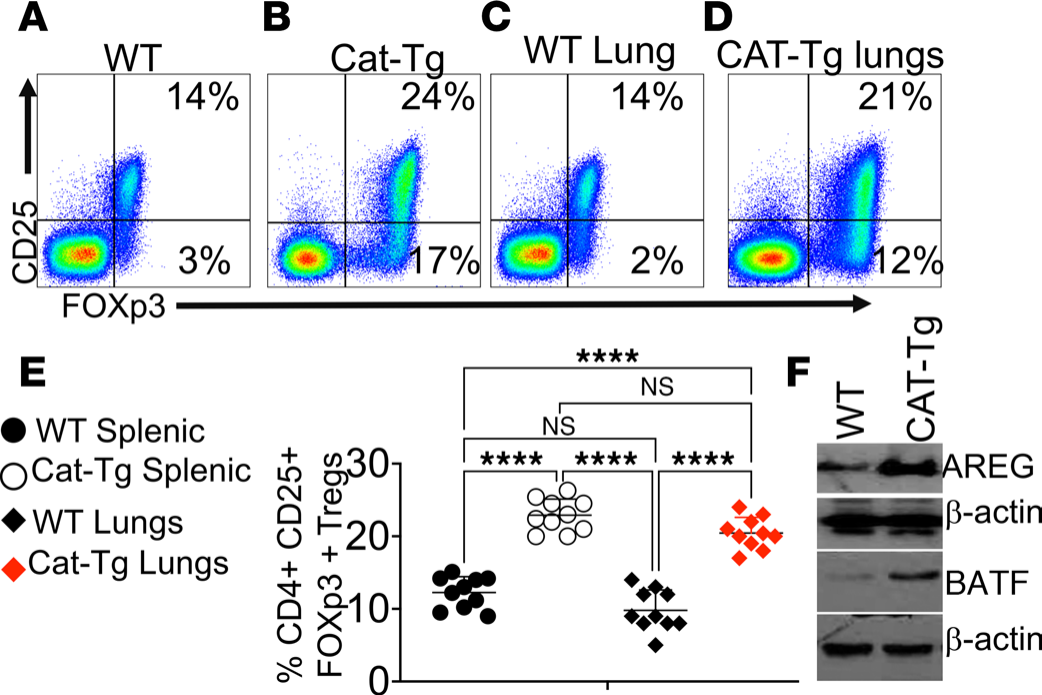
β-Catenin stabilization increases Tregs and enhances AREG and BATF expression. (**A** and **B**) Flow cytometry of splenocytes from WT and CAT-Tg mice. Cells were gated on CD3^+^CD4^+^ T cells and analyzed for CD25 and intracellular FOXP3 expression. (**C** and **D**) Lung leukocytes from WT and CAT-Tg mice were analyzed for Tregs using the same gating strategy (CD3^+^CD4^+^CD25^+^FOXP3^+^). (**E**) Representative flow plots and quantification of CD4^+^CD25^+^FOXP3^+^ Treg frequencies. (**F**) CD25^+^FOXP3^+^ Tregs were FACS-sorted from lung tissue and analyzed by immunoblot for AREG and BATF, with β-actin as a loading control. Data are presented as mean ± SEM; sample sizes (*n* = 15–25 mice per group) are indicated in panels. Each experiment was repeated 3 times. Statistical significance was determined using 2-tailed Student’s *t* test, 1-way ANOVA, or 2-way ANOVA as appropriate; **P < 0.01, ***P < 0.001, ****P < 0.0001.

The role of Tregs in ameliorating AH has not been well defined. Tregs are established mediators of immune suppression and tissue repair, acting through the release of antiinflammatory cytokines such as IL-10 to limit neutrophil and inflammatory monocyte activity and to restore immune balance after injury (58, 59). In addition, Tregs directly promote tissue repair after inflammatory damage (58, 60). To determine whether β-catenin stabilization affects Tregs, splenocytes and lungs from WT and CAT-Tg mice were analyzed by flow cytometry. CD3^+^CD4^+^ T cells were gated and assessed for CD25 and FOXP3 expression. CAT-Tg mice exhibited a significantly increased frequency of Tregs, including both conventional CD25^+^FOXP3^+^ and noncanonical CD25^–^FOXP3^+^ subsets (30, 61), compared with WT controls (Figure 5, A–E). These data suggest that β-catenin stabilization promotes Treg expansion during AH. To further define Treg-mediated protective mechanisms, we examined Areg, an epidermal growth factor receptor ligand that promotes tissue repair independently of classical Treg suppressive functions (16, 62–64). CD25^+^FOXP3^+^ Tregs were FACS-sorted from the lungs of WT and CAT-Tg mice using FOXP3–RFP reporter expression (Figure 5, C and D), and Areg expression was assessed by immunoblotting. Tregs from CAT-Tg mice expressed substantially higher levels of Areg than WT controls (Figure 5F), indicating enhanced tissue-repair capacity. Consistent with this phenotype, CAT-Tg Tregs also expressed significantly higher levels of the transcription factor BATF, which is required for Treg homeostasis, differentiation, and stability (65) (Figure 5F). BATF sustains FOXP3 expression, and these findings indicate that β-catenin stabilization not only expands Treg populations but also reinforces their lineage stability and suppressive function, thereby limiting uncontrolled inflammation. Collectively, these results demonstrate that β-catenin stabilization programs a protective, lineage-stable Treg phenotype with enhanced expansion and tissue-repair capacity. We next tested the functional consequences of CAT-Tg–derived Tregs in vivo.

### Adoptive transfer of CAT-Tg Tregs rescues AH in vivo.

**Figure 6:**
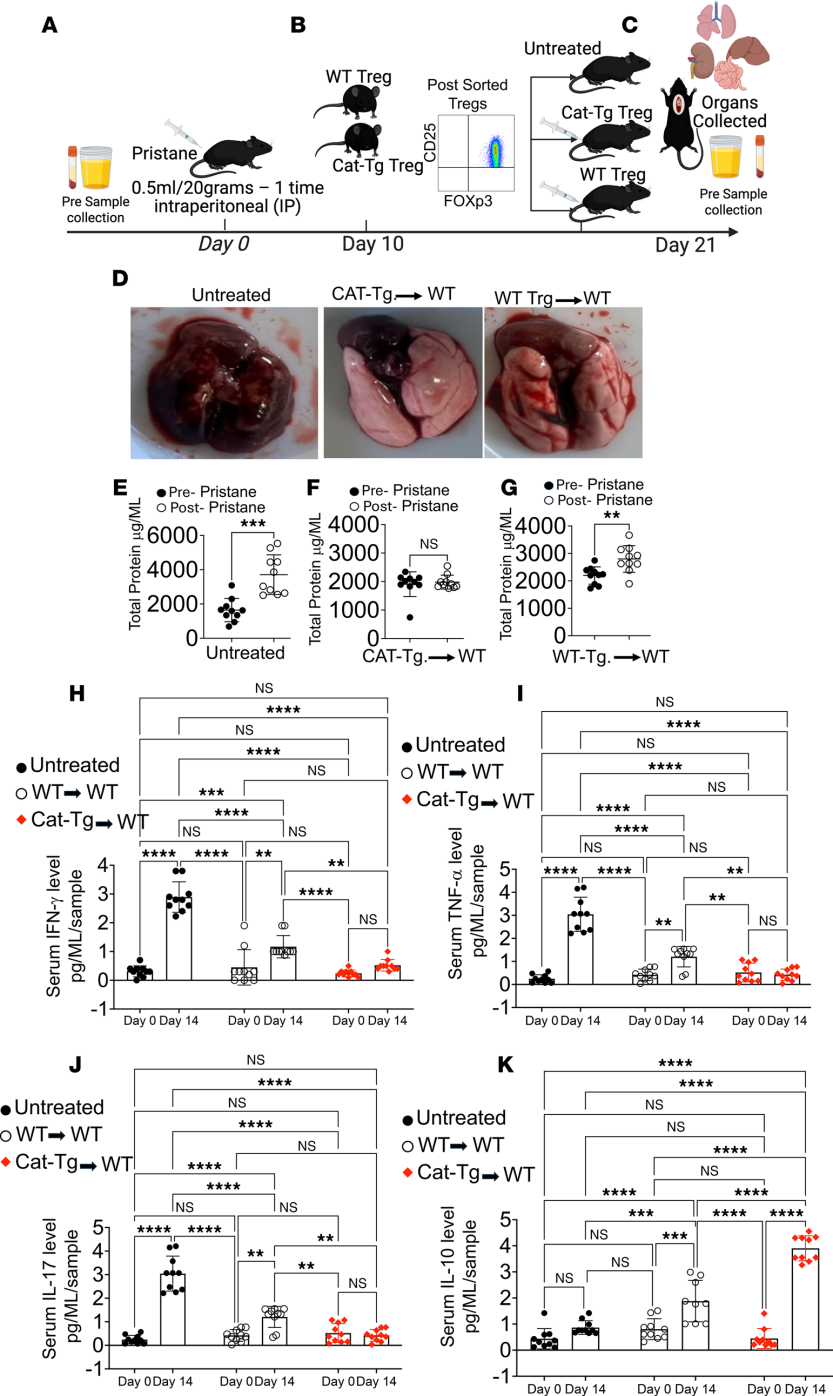
Adoptive transfer of CAT-Tg Tregs rescues pristane-induced alveolar hemorrhage in vivo. (**A**) Experimental schematic. WT recipient mice were assigned to 3 groups, and baseline urine and blood were collected. (**B**) CD25^+^FOXP3^+^ Tregs were isolated from WT or CAT-Tg donor mice by flow cytometry. (**C**) Recipients were left untreated or received 1 × 10^6^ WT Tregs or 1 × 10^6^ CAT-Tg Tregs by adoptive transfer. (**D**) Representative gross lung images from pristane-injected WT mice (untreated) and from mice receiving WT or CAT-Tg Tregs. (**E**–**G**) Urinary protein was quantified by BCA assay before and after pristane challenge. (**H**–**K**) Serum cytokines (IFN-γ, TNF-α, IL-17, and IL-10) were quantified in each group at baseline and after pristane challenge (Figure 4I). Data are presented as mean ± SEM; sample sizes (*n* = 15–25 mice per group) are indicated in panels. Each experiment was repeated 3 times. Statistical significance was determined using 2-tailed Student’s *t* test or 2-way ANOVA as appropriate; **P < 0.01, ***P < 0.001, ****P < 0.0001.

To determine whether Tregs from CAT-Tg mice, characterized by elevated Areg and BATF expression, confer protection against AH in vivo, we performed adoptive transfer experiments. WT mice were divided into 3 groups: untreated controls, recipients of WT Tregs, and recipients of CAT-Tg Tregs. Baseline serum and urine samples were collected prior to pristane administration. AH was induced by pristane injection, and disease was allowed to establish for 10 days. At day 10 after pristane injection, splenic CD3^+^CD4^+^CD25^+^FOXP3^+^ Tregs were FACS-purified from WT or CAT-Tg donor mice and adoptively transferred (1 × 10^6^ cells per mouse) into WT recipients, while one cohort remained untreated. At day 21 after pristane injection, lungs, serum, and urine were collected for analysis (Figure 6, A–D). Gross pathological examination revealed severe AH in untreated pristane-injected WT mice (Figure 6D). In contrast, mice receiving Tregs from CAT-Tg mice were fully protected, with lungs appearing grossly normal, whereas recipients of WT Tregs exhibited only partial protection. Consistent with lung pathology, proteinuria was markedly elevated in untreated and WT Treg–recipient mice compared with baseline levels, whereas adoptive transfer of CAT-Tg Tregs completely prevented proteinuria (Figure 6, E–G). Together, these findings demonstrate that CAT-Tg Tregs confer superior protection against both pulmonary and renal injury during AH. Cytokine analyses further demonstrated the superior protective capacity of CAT-Tg Tregs. WT mice receiving CAT-Tg Tregs exhibited significantly reduced levels of IFN-γ and TNF-α compared with mice receiving WT Tregs (Figure 6, H and I). IL-17 levels were unchanged in CAT-Tg Treg recipients, whereas WT Tregs only modestly reduced IL-17 relative to untreated mice, which remained significantly higher than levels observed in CAT-Tg Treg recipients (Figure 6J). Importantly, adoptive transfer of CAT-Tg Tregs induced a robust increase in IL-10, a key antiinflammatory cytokine, compared with both untreated and WT Treg–treated mice (Figure 6K). Together, these findings indicate that β-catenin stabilization enhances Treg-mediated protection against AH by suppressing proinflammatory cytokines while promoting IL-10 production.

To assess the persistence and tissue trafficking of donor Tregs, adoptive transfer experiments were repeated using congenic CD45.2 WT recipients and CD45.1 donor mice. At day 21 after transfer, both donor- and host-derived Tregs were detected in the spleen (Supplemental Figure 3, A–B). IHC analysis of lung tissue identified donor Tregs within inflamed lungs, confirmed by colocalization of CD4 (green), donor CD45.1 (blue), and FOXP3 (red) signals (Supplemental Figure 2A). These data confirm engraftment and lung homing of donor CAT-Tg Tregs. Collectively, these results demonstrate that CAT-Tg Tregs, enriched for AREG and BATF, confer superior protection against AH compared with WT Tregs by suppressing proinflammatory cytokines, enhancing IL-10 production, and efficiently trafficking to sites of lung inflammation.

### β-Catenin agonists recapitulate the protective effects of genetic stabilization.

**Figure 7:**
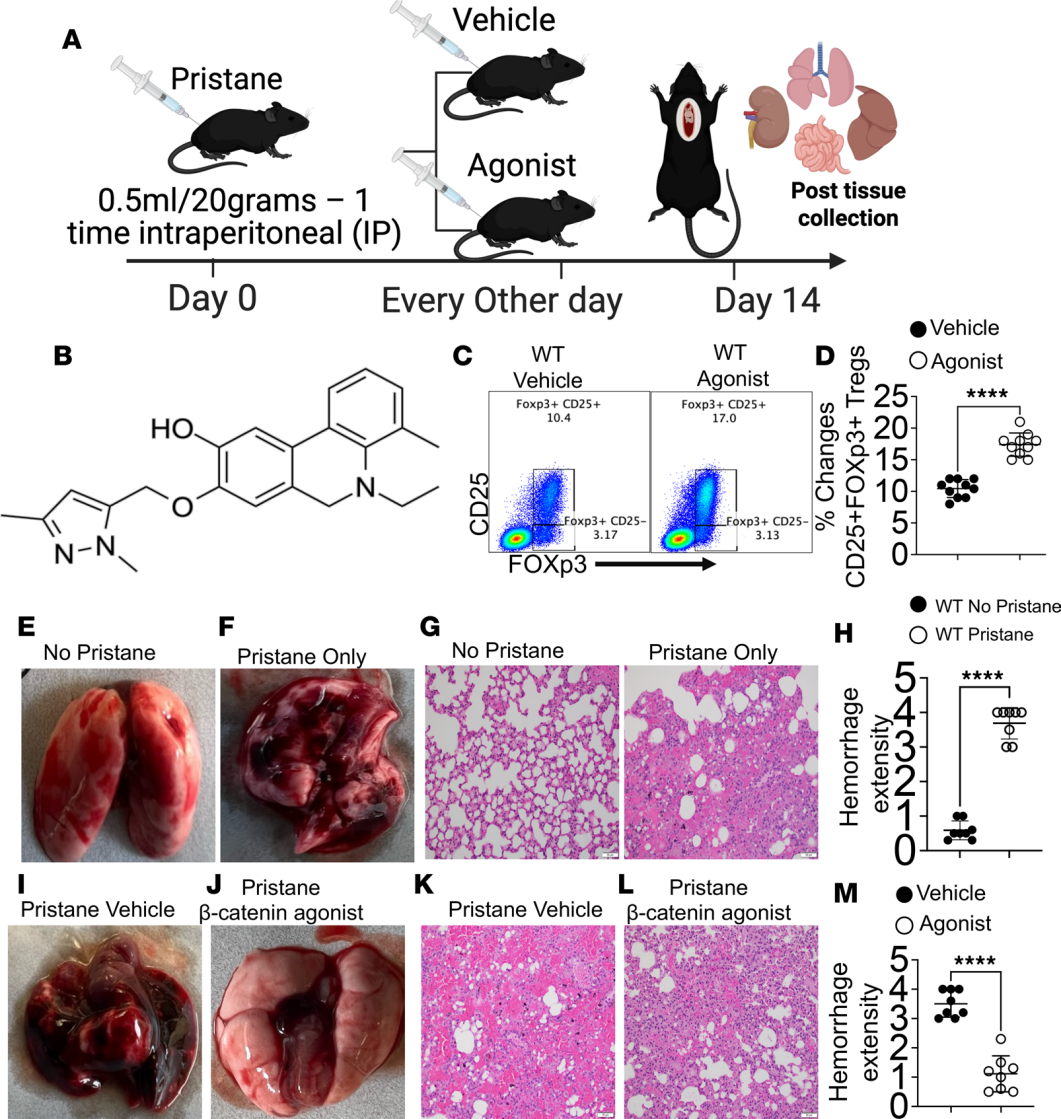
β-Catenin agonist treatment phenocopies genetic β-catenin stabilization and protects against pristane-induced alveolar hemorrhage. (**A**) Experimental schematic. WT mice were injected with pristane to induce alveolar hemorrhage (AH) and assigned to treatment groups as indicated. (**B**) Chemical structure of the β-catenin agonist. (**C** and **D**) Representative flow plots and quantification of lung Tregs (CD4^+^CD25^+^FOXP3^+^) in WT mice treated with vehicle or β-catenin agonist; lungs were harvested on day 14. (**E** and **F**) Gross and histological comparison of untreated WT controls and pristane-only WT mice (no vehicle or agonist). (**G** and **H**) Representative H&E-stained lung sections and corresponding pathology scores for cohorts in **E** and **F**. (**I** and **J**) Gross and histological comparison of pristane-injected WT mice treated with vehicle versus β-catenin agonist (10 μg per 20 g body weight). (**K**–**M**) Representative H&E-stained lung sections and quantitative pathology scoring for cohorts in **I** and **J**. Data are presented as mean ± SEM; sample sizes (*n* = 15–25 mice per group) are indicated in panels. Each experiment was repeated 3 times. Statistical significance was determined using the appropriate test; **P < 0.01, ***P < 0.001, ****P < 0.0001. Scale bar:50 μm.

To determine whether pharmacological activation of β-catenin can mimic genetic stabilization in vivo, we treated WT mice with Wnt/β-catenin agonists (MedChemExpress; HY-114321) (66–68), in a pristane-induced AH model. WT mice were injected with pristane, and urine and blood were collected for baseline and posttreatment analyses. Mice received either vehicle (DMSO) or a β-catenin agonist (10 μg per 20 g body weight) twice weekly for 14 days (Figure 7, A and B). Animals were euthanized on day 14, and lungs were harvested for analysis. Treatment with β-catenin agonists significantly increased the frequency of FOXP3^+^ Tregs in the lungs compared with vehicle-treated controls (Figure 7, C and D). As expected, pristane-injected WT mice exhibited severe lung injury relative to untreated controls, with extensive AH confirmed by H&E staining (Figure 7, D–H). In contrast, WT mice treated with the β-catenin agonist showed minimal lung damage despite pristane challenge, whereas vehicle-treated mice displayed substantial hemorrhage (Figure 7, I and J). Quantitative assessment demonstrated significantly reduced red blood cell accumulation and tissue injury in the β-catenin agonist–treated group (Figure 7, K–M). Collectively, these findings demonstrate that pharmacological activation of β-catenin phenocopies genetic stabilization, protecting against pristane-induced AH by promoting a suppressive Treg-mediated immune program.

### β-Catenin agonists reduce proteinuria and proinflammatory cytokines in the AH model.

**Figure 8:**
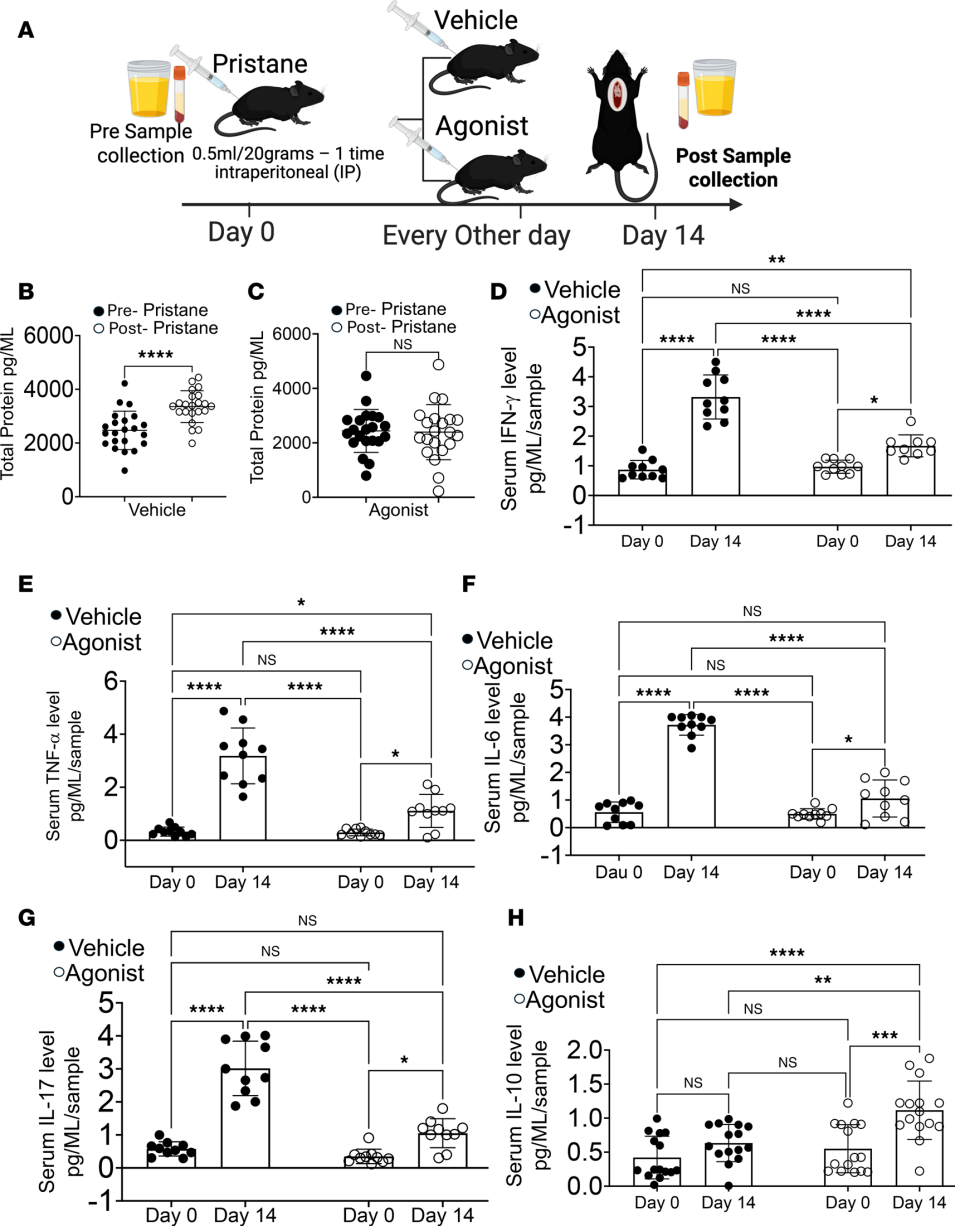
β-Catenin agonists reduce proteinuria and modulate cytokine responses in the AH model. (**A**) Experimental schematic. Urine and blood were collected at baseline (pre-pristane). WT mice were injected with pristane to induce alveolar hemorrhage (AH) and treated with vehicle (DMSO) or β-catenin agonist (dose as indicated). On day 14, mice were euthanized and urine and serum were collected. (**B**–**D**) Urinary protein was quantified by BCA assay, with comparisons between pre- and post-pristane samples within each group. (**E**–**H**) Serum cytokines (IFN-γ, TNF-α, IL-6, IL-18, and IL-10) were quantified by multiplex bead-based assay and compared across groups and time points as indicated. Data are presented as mean ± SEM; sample sizes (*n* = 15–25 mice per group) are indicated in panels. Each experiment was repeated 3 times. Statistical significance was determined using 2-tailed Student’s *t* test or 2-way ANOVA as appropriate; **P < 0.01, ***P < 0.001, ****P < 0.0001.

To determine whether pharmacological β-catenin activation suppresses inflammatory responses during AH, AH was induced as described above (Figure 7 and Figure 8A). Urine analysis revealed that WT mice injected with pristane and treated with vehicle developed significantly increased proteinuria compared with pre-pristane baseline levels. In contrast, WT mice treated with β-catenin agonists exhibited no significant change in proteinuria before versus after pristane injection, indicating protection from renal injury (Figure 8, B and C). Consistent with these findings, β-catenin agonist–treated mice displayed significantly reduced serum levels of IFN-γ and TNF-α compared with untreated or vehicle-treated controls. However, cytokine suppression was incomplete and did not reach the magnitude observed with genetic β-catenin stabilization (Figure 8, D and E). Similarly, levels of IL-6 and IL-17 were reduced after β-catenin agonist treatment, albeit to a lesser extent than in the genetic model (Figure 8, F and G). Notably, β-catenin agonist treatment significantly increased IL-10 production relative to untreated and vehicle-treated controls (Figure 8H). In contrast, no significant differences were observed in IL-12 or IL-13 levels (Supplemental Figure 4). These data showed that pharmacological β-catenin activation amplifies Treg-associated antiinflammatory responses while suppressing proinflammatory cytokine production, supporting its therapeutic potential for AH.

### β-Catenin stabilization regulates gene expression during AH.

**Figure 9:**
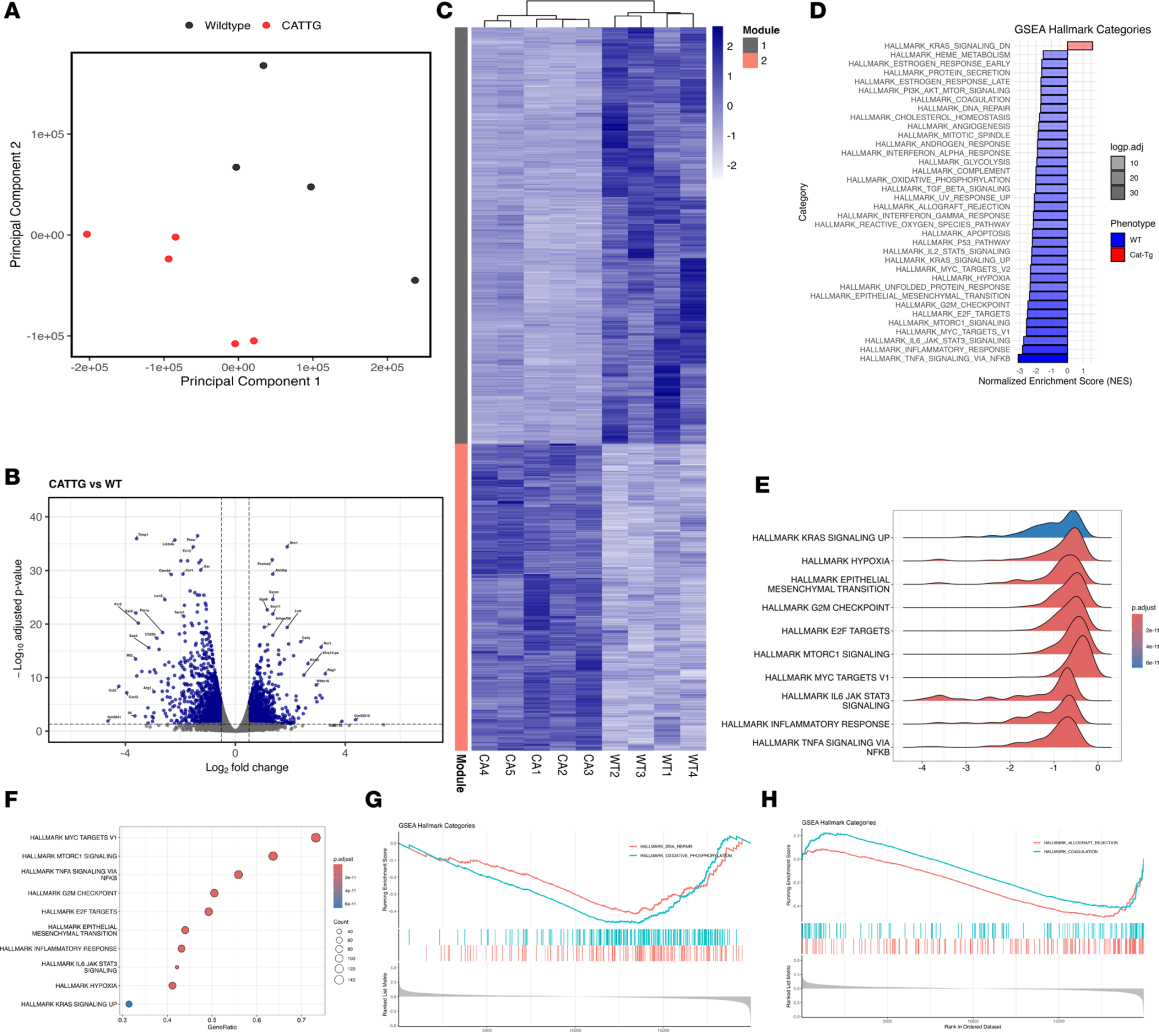
β-Catenin stabilization reprograms lung gene expression during pristane-induced alveolar hemorrhage. (**A**) Principal component analysis (PCA) showing separation of WT and CAT-Tg lung transcriptomes at day 14 after pristane-induced AH (*n* = 4 per group). (**B**) Volcano plot of differentially expressed genes (DEGs; FDR ≤ 0.05, |log_2_FC| ≥ 0.5) between CAT-Tg and WT lungs; positive log_2_FC indicates higher expression in CAT-Tg. (**C**) Hierarchical clustering heatmap of row-scaled normalized expression for DEGs across all samples (*n* = 4 per group). (**D**) Summary of Hallmark pathway enrichment by GSEA, highlighting pathways enriched in WT versus CAT-Tg lungs following AH. (**E**) Ridge plot showing normalized enrichment score (NES) distributions for selected Hallmark gene sets. (**F**) Dot plot summarizing gene ratios for selected Hallmark gene sets; dot size reflects the number of genes contributing to enrichment. (**G** and **H**) Representative GSEA enrichment plots for selected Hallmark pathways, including DNA repair and oxidative phosphorylation (**G**) and allograft rejection and coagulation (**H**).

Our findings demonstrate that β-catenin stabilization protects the lung from AH and promotes expansion of Tregs expressing Areg and BATF, which contribute to tissue repair and suppression of inflammatory cytokines. However, the global transcriptomic programs regulated by β-catenin stabilization during AH remained undefined. To address this, AH was induced with pristane as described above, and lung tissues were harvested at day 14 for bulk RNA-Seq. Principal component analysis (PCA) revealed clear segregation between WT and CAT-Tg samples (PC1: 53.05%, PC2: 25.60%, PC3: 12.05%) (Figure 9A). Differential expression analysis identified 2,688 genes (FDR ≤ 0.05, |log_2_FC| ≥ 0.5), with 1,464 genes downregulated and 1,224 genes upregulated in CAT-Tg lungs compared with WT controls (Figure 9B). Hierarchical clustering of normalized expression values separated these genes into 2 modules: module 1, comprising genes downregulated in CAT-Tg samples, and module 2, comprising genes upregulated in CAT-Tg samples (Figure 9C). Gene Ontology (GO) analysis of module 1 revealed enrichment of pathways related to stress responses, immune activation, and inflammation. In contrast, module 2 was enriched for pathways associated with cell projection assembly, cell motility, and tissue organization. Gene set enrichment analysis (GSEA) using Hallmark pathways from MSigDB (73) demonstrated broad enrichment of inflammatory, stress-response, metabolic, and cell-cycle pathways — including TNF-α/NF-κB, IL-6/JAK/STAT3, IFN-γ response, oxidative stress, coagulation, and hypoxia — in WT lungs relative to CAT-Tg samples (Figure 9, D–H). Conversely, KRAS signaling (down) was selectively enriched in CAT-Tg lungs. Collectively, these data demonstrate that β-catenin stabilization rewires lung transcriptional programs during AH, suppressing inflammatory and stress-response pathways while activating cell motility and tissue-repair programs, thereby establishing a protective and regenerative immune environment.

### β-Catenin agonists affect gene expression during AH.

**Figure 10:**
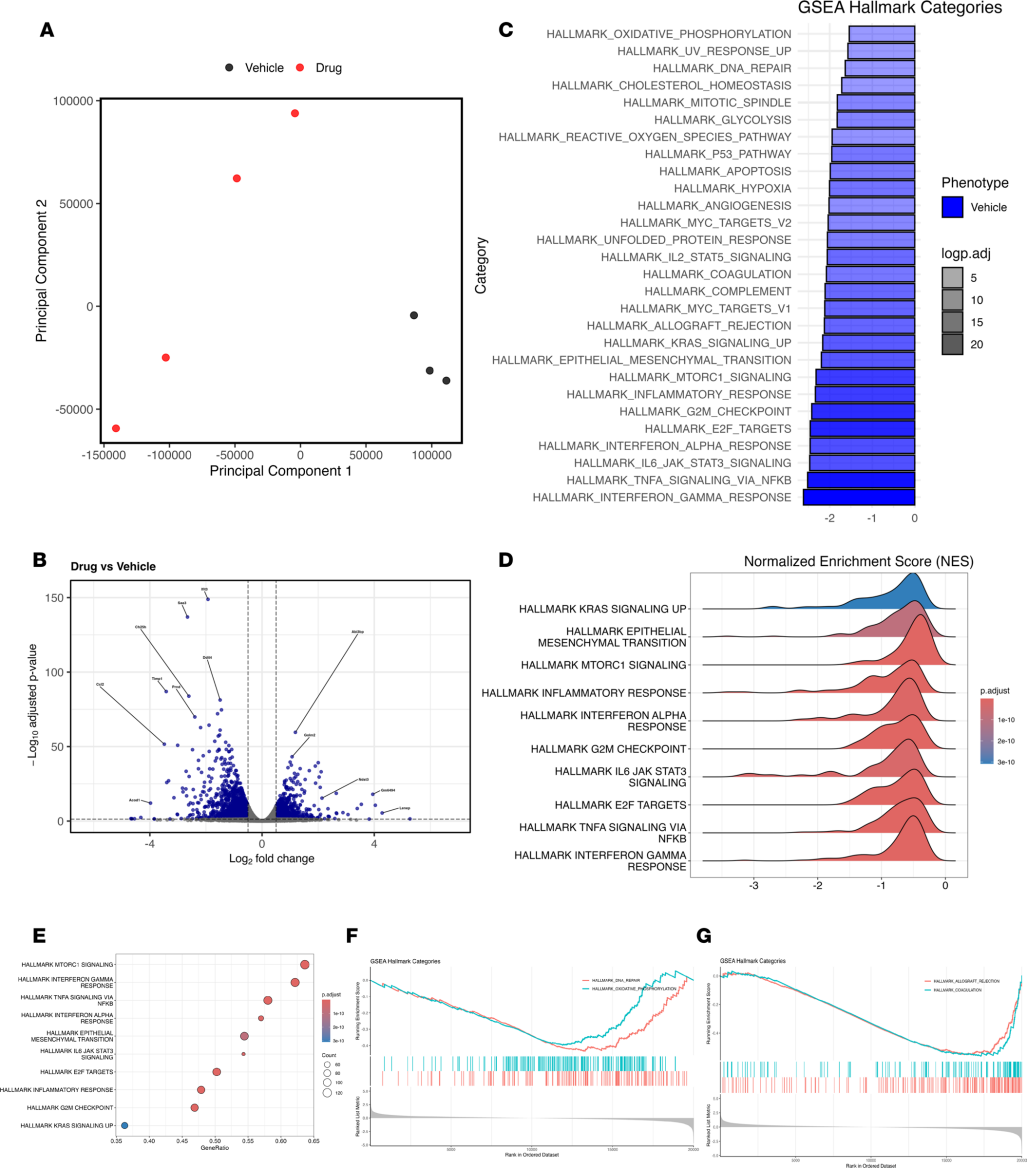
β-Catenin agonist treatment reprograms lung gene expression during pristane-induced alveolar hemorrhage. (**A**) Principal component analysis (PCA) showing separation of vehicle- and β-catenin agonist–treated lung transcriptomes at day 14 after pristane-induced AH (*n* = 3 vehicle, *n* = 4 agonist). (**B**) Volcano plot of differentially expressed genes (DEGs; FDR ≤ 0.05, |log_2_FC| ≥ 0.5) between agonist- and vehicle-treated lungs; positive log_2_FC indicates higher expression in agonist-treated samples. (**C**) Summary of Hallmark pathway enrichment by GSEA, highlighting pathways enriched in vehicle versus agonist-treated lungs. (**D**) Ridge plot showing normalized enrichment score (NES) distributions for selected Hallmark gene sets. (**E**) Dot plot summarizing gene ratios for selected Hallmark gene sets; dot size reflects the number of genes contributing to enrichment. (**F** and **G**) Representative GSEA enrichment plots for selected Hallmark pathways, including DNA repair and oxidative phosphorylation (**F**) and allograft rejection and coagulation (**G**).

To determine whether pharmacological β-catenin activation recapitulates the transcriptomic effects of genetic stabilization during AH, WT mice were subjected to pristane-induced AH and treated with either vehicle or a β-catenin agonist, as described above. At day 14, lungs were harvested for bulk RNA-Seq. Differential expression analysis identified 2,565 genes (μμμFDR ≤ 0.05, |log_2_FC| ≥ 0.5) between agonist- and vehicle-treated lungs, with 1,583 genes downregulated and 982 genes upregulated in agonist-treated samples (Figure 10B). PCA demonstrated clear separation between agonist- and vehicle-treated groups (PC1: 65.84%, PC2: 20.22%, PC3: 6.82%) (Figure 10A). GO enrichment analysis of downregulated genes (module 1) revealed pathways associated with immune activation, stress responses, and proliferative signaling, including BCR stimulation, immune system processes, E2F-associated complexes, and cell cycle–related pathways. In contrast, upregulated genes (module 2) were enriched for pathways related to cell projection assembly, microtubule organization, cilium-dependent motility, and cytoskeletal remodeling. GSEA using Hallmark pathways demonstrated significant negative enrichment of inflammatory, stress-response, and proliferative programs in agonist-treated lungs, including IFN-γ response, TNF-α/NF-κB signaling, IL-6/JAK/STAT3 signaling, transcription factor E2F targets, G2M checkpoint, mTORC1 signaling, epithelial-mesenchymal transition, and oxidative phosphorylation (Figure 10, C–G). These findings show that pharmacological β-catenin activation recapitulates the transcriptomic reprogramming observed with genetic stabilization by suppressing inflammatory and stress-response pathways while enhancing motility- and repair-associated programs, supporting a mechanistic basis for therapeutic protection during AH.

## Discussion

AH is a life-threatening condition marked by severe pulmonary inflammation and high mortality (69–73). Despite its clinical impact, the immunological mechanisms by which Tregs mitigate lung injury in AH remain incompletely defined. We previously showed that T cells from CAT-Tg mice mediate potent antitumor immunity without inducing graft-versus-host disease (GVHD) in an allogeneic transplant model (36). In that context, CAT-Tg T cells displayed an activated phenotype characterized by expanded CM and EM memory subsets and increased expression of CD44, CD122, Eomes, and T-bet (Figures 1 and 2).

In this study, we show that stabilization of β-catenin under the proximal Lck promoter (23) recapitulates and extends these immunological features in inflammatory lung injury. We found that β-catenin stabilization expanded Tregs, resembling the phenotype of T cells with attenuated TCR signaling — an immune-regulatory state rather than a pathogenic one. Although β-catenin has been implicated in thymocyte development (23), our data extend this role to the periphery, establishing β-catenin as a regulator of T cell activation, lineage stability, and tissue-protective function.

Using a pristane-induced murine AH model, we show that constitutive β-catenin expression confers robust protection against lung injury. CAT-Tg mice displayed markedly reduced hemorrhage and preserved lung architecture compared with WT controls. No significant differences were observed in liver, kidney, or spleen pathology within the first 14 days, suggesting an early, lung-focused protective effect. However, WT mice developed early proteinuria and later renal and splenic pathology (4–6 weeks), indicating that β-catenin signaling may also influence systemic disease progression over time. Our data show that β-catenin stabilization reduces phosphorylation of STAT1, STAT3, and JAK1 — central nodes in cytokine signaling downstream of IFN-γ, IL-6, TNF-α, IL-18, IL-17, and IL-4 (30, 42). Consistent with this signaling restraint, CAT-Tg mice exhibited markedly lower proinflammatory cytokines (IFN-γ, IL-6, TNF-α, IL-18, IL-17, and IL-5) during AH, alongside increased antiinflammatory cytokines including IL-4, IL-13, and IL-10 (74, 75). Collectively, these findings position β-catenin as a key regulator of cytokine storm severity and a potential therapeutic target for AH and other cytokine-driven inflammatory diseases.

Tregs are central to immune homeostasis and help suppress inflammation and cytokine storm–driven pathology (30, 76). However, induced Tregs often lose FOXP3 expression and lineage stability during in vitro expansion (77, 78), whereas naturally occurring Tregs are present at low frequencies in vivo. Notably, CAT-Tg mice exhibited a marked expansion of Tregs relative to WT controls, with increased accumulation in the lung during AH (Figure 5, A–E). To define the mechanisms underlying this protective response, we focused on AREG and the transcription factor BATF, key regulators of tissue repair and immune stability (17, 65, 79–83). β-Catenin stabilization activates transcriptional programs that include direct induction of AREG (9, 12); consistent with this finding, CAT-Tg lungs expressed elevated AREG levels and were protected from AH (11, 13). Functional relevance was supported by adoptive transfer experiments in which CAT-Tg–derived Tregs conferred robust protection, suppressing IFN-γ, TNF-α, and IL-17 while enhancing IL-10 production (Figure 6). Collectively, these findings link β-catenin stabilization to Treg expansion and stability and to tissue repair during AH. Because genetic models are not always directly translatable, we next evaluated pharmacological strategies to activate β-catenin during AH. Treatment of WT mice with β-catenin agonists increased Treg frequencies, reduced proinflammatory cytokine production, and enhanced IL-10 expression, conferring substantial protection against AH. Pharmacological β-catenin activation largely recapitulated the protective effects of genetic β-catenin stabilization. These findings support therapeutic targeting of β-catenin in AH and potentially other cytokine storm–mediated inflammatory diseases. RNA-Seq analyses showed that β-catenin stabilization reprograms lung transcriptional landscapes during AH by suppressing inflammatory and stress-response pathways while activating cell motility and tissue-repair programs, thereby promoting a protective, regenerative environment. Pharmacological β-catenin activation closely mirrored these transcriptomic effects, attenuating inflammatory, stress-response, and proliferative signaling while enhancing ciliary and motility-associated pathways. Collectively, these findings establish β-catenin stabilization as a central regulator of protective transcriptional programs during AH, with therapeutic potential to suppress inflammation and promote tissue repair.

## Methods

### Sex as a biological variable.

Sex was not considered as a biological variable in this report because the study was designed specifically to investigate mechanisms of acute AH. In this model, male mice consistently develop acute AH, while female mice typically exhibit a more chronic AH course, which represents a biologically distinct phenotype with different kinetics. To ensure a consistent acute disease trajectory and to minimize variability introduced by sex-dependent differences in disease course, we restricted the study to male mice.

### Mice.

We used both male and female mice, but female mice did not show severe AH. The mice used in these studies were all male. CAT-Tg mice have been previously described (24, 25) and were provided in-house. C57BL/6 mice were purchased from Charles River Laboratories or The Jackson Laboratory. Mice aged 8–12 weeks were used for all experiments, with age- and sex-matched controls included throughout.

### Reagents, cell lines, flow cytometry.

Monoclonal antibodies were purchased from BioLegend or eBioscience and BD Pharmingen. All flow cytometry antibodies were used at a 1:100 dilution unless otherwise indicated. Antibodies included anti-mouse CD3 (catalog 100102), anti-CD28 (catalog 102116), CD3-BV605 (catalog 100237), CD4-PE (catalog 100408), CD8-PE/Cy7(catalog 100722), Eomes-PE/Cy7 (catalog 25-4877-42), CD44–Pacific Blue (catalog 156006), CD122-APC (catalog 105912), CD62L-APC/Cy7(catalog 304814), T-bet–BV421(catalog 644816), TNF-α–FITC (catalog 502906), IFN-γ–APC (catalog 505810), TCF-1–PE (catalog 564217), FOXP3–Pacific blue (catalog 126410), CD25–FITC (catalog 101908), CD11b-FITC (catalog 101205), and Ly-6C-APC (catalog 128016). Flow cytometry was performed on an LSRFortessa (BD Biosciences). Dead cells were excluded using a Live/Dead viability dye (BioLegend), and singlets were identified by forward and side-scatter properties. CD4^+^ and CD8^+^ T cells were gated from live CD3^+^ T cells, and surface and intracellular markers (CD44, CD62L, T-bet, Eomes, TCF-1, FOXP3, and cytokines) were analyzed within these subsets. Data were analyzed using FlowJo (Tree Star) as previously described (30, 61).

### Consumables.

All consumables were purchased from eIMMUNA medical supply.

### Proteinuria assays.

Urine was collected on day 0 (pre-pristane) and on days 14 or 21 after pristane injection. Total urinary protein was quantified using a Pierce BCA protein assay kit (Thermo Fisher Scientific; 23225). Urine was diluted 1:10 in PBS (3 μL urine + 27 μL PBS) and mixed with BCA working reagent (50:1, reagent A/reagent B). Diluted urine (25 μL) was combined with working reagent (200 μL) in a 96-well plate, briefly centrifuged (30 seconds at 135*g*), and incubated at 37°C for 30 minutes. Absorbance was measured at 562 nm using a BioTek FL ×800 plate reader, and protein concentrations (μg/mL) were calculated in Gen5 using a BSA standard curve.

### Cytokine production assays.

Serum was collected from cardiac blood at baseline (pre-pristane) and on day 14 after treatment. Cytokine concentrations — including IFN-γ, TNF-α, IL-5, IL-12, IL-6, IL-10, IL-9, IL-17A, IL-17F, IL-22, and IL-13 — were quantified using a multiplex bead-based immunoassay (LEGENDplex, BioLegend; 741011) according to the manufacturer’s instructions (37, 38). Samples were acquired on an LSRFortessa (BD Biosciences), and cytokine concentrations were calculated using LEGENDplex data analysis software (BioLegend).

### Western blotting.

For STAT and JAK signaling, T cells were isolated from either WT or Cat-Tg mice using either CD4 beads (130-114-043) or CD8 beads (130-117-044) from Miltenyi Biotec. Cell were stimulated with precoated plates with purified CD3 (catalog 100202) and CD28 (catalog 122022), both from BioLegend. Cells were stimulated for 5 minutes. For Western blot data in Figure 5F, Tregs from either WT or CAT-Tg were sorted as described below. Cells in both cases were lysed in freshly prepared RIPA buffer (Thermo Fisher Scientific; PI9900) supplemented with a complete protease inhibitor cocktail (Sigma-Aldrich; 11697498001) and clarified by centrifugation at 1,840*g* for 10 minutes at 4°C. Lysates equivalent to 1 × 10^6^ cells per sample were resolved on 12%–18% SDS-PAGE and transferred to nitrocellulose membranes for immunoblotting. Unless otherwise specified, reagents were obtained from Invitrogen or Sigma-Aldrich. For signaling analyses, membranes were probed with antibodies against phospho-STAT1 (catalog 9167), total STAT1 (catalog 9172), phospho-STAT3 (catalog 9131), JAK1 (catalog 3332), phospho-JAK3 (catalog 5031), and β-actin (catalog 4970) (all from Cell Signaling Technology). Additional antibodies included AREG (clone AREG559, Thermo Fisher Scientific; 14-9999-82) and BATF (mouse monoclonal, Thermo Fisher Scientific; MA5).

### Histopathological evaluation.

Mice were injected with pristane and euthanized on days 14 or 21 after treatment. Lungs, liver, kidneys, and small intestine were harvested; fixed in 10% neutral-buffered formalin; and processed for paraffin embedding, sectioning, and H&E staining by the Histology Core Facility at SUNY Upstate Medical University. AH pathology was evaluated by a board-certified pathologist blinded to experimental group and disease status. Tissue injury was graded using established criteria (84, 85), and histopathology scores were analyzed using the Mann-Whitney *U* test.

### Fluorescence microscopy and histologic analysis.

Inflammatory monocyte infiltration was assessed 14 days after AH induction in C57BL/6 WT and CAT-Tg mice. Circulating blood was cleared by gentle cardiac perfusion with 2 mL warm PBS using a 25-gauge needle, followed by tracheal inflation with a 1:1 mixture of PBS and OCT compound to preserve alveolar architecture. Lungs were excised, embedded in OCT, flash-frozen, and cryo-sectioned at 10 μm. Sections were fixed in acetone (5 minutes, room temperature), blocked for endogenous biotin, and stained overnight at 4°C for CD11b^+^ (14-0112-82) and Ly6C (MA1-81899) monocytes (ThermoFisher Scientific). Images were acquired using a Zeiss Axioplan microscope with AxioVision 4.8 software. Histological scoring was performed by a board-certified pathologist blinded to experimental groups. For IHC, antibodies included anti-CD4 (clone GK1.5, Thermo Fisher Scientific; 14-0041-82), anti-CD45.2 (Thermo Fisher Scientific; 14-0454-82), and anti-FOXP3 (Thermo Fisher Scientific; 14-5773-82). Avidin biotin blocking kit (Thermo Fisher Scientific; 001303), rat IgG2a isotype control (Thermo Fisher Scientific; PA533213), streptavidin Alexa Fluor 594 (Thermo Fisher Scientific; S11227), DAPI (Thermo Fisher Scientific; 62247), Alexa Fluor 488 (CD11b mAb M1/70) Bioscience (Invitrogen; 53-0112-80), Ly-6C mAb (Invitrogen; MAI 81899), goat anti-mouse IgG (H+L) and secondary antibody Alexa Fluor 647 (A32728TR; Invitrogen).

### Isolation of Tregs.

CAT-Tg and WT mice were bred onto the C57BL/6 background and crossed with the Foxp3tm1Flv/J strain, an X-linked knockin reporter in which Foxp3-expressing cells are co-marked with monomeric red fluorescent protein (mRFP). In this model, mRFP faithfully reports endogenous Foxp3 expression in lymphocytes. The Foxp3^tm1Flv/J strain was provided by Avery August (Cornell University), and Foxp3-RFP expression was confirmed in both WT and CAT-Tg mice. CD4^+^ lymphocytes were isolated from spleens using anti-CD4 magnetic microbeads and column-based separation (Miltenyi Biotec; 130-114-043). Tregs were identified as CD3^+^CD25^+^Foxp3(RFP^+^) cells and purified by FACS on a FACSAria III (BD Biosciences). Sorted Treg purity routinely exceeded 98% unless otherwise specified. Unless noted, reagents and cell culture materials were obtained from Sigma-Aldrich or Invitrogen, consistent with published protocols (30, 61).

### RNA-Seq.

For genetic β-catenin stabilization studies, 4 WT and 4 CAT-Tg mice were injected with pristane and euthanized on day 14. Lungs were harvested and snap-frozen. For pharmacological studies, WT mice were injected with pristane and subsequently treated with a β-catenin agonist as described above, after which lungs were collected. Samples from both studies were processed by the Molecular Analysis Core Facility at SUNY Upstate Medical University for RNA extraction, library preparation, and high-throughput sequencing. RNA-Seq datasets included WT, CAT-Tg, and vehicle-treated versus agonist-treated WT mice after pristane challenge. Data processing and analysis were performed in R (v4.5.1) using RStudio (v2025.05.1+513) and Bioconductor packages. Transcript abundance was quantified by pseudoalignment with Kallisto (v0.51.1) (98), and transcript-per-million (TPM) values were normalized across samples and modeled using the *sleuth* package (99, 100). Differentially expressed genes were defined by |log_2_ fold change| of 0.5 or greater and FDR of 0.05 or less after Benjamini-Hochberg correction (101). Differentially expressed gene sets were used for hierarchical clustering and heatmap generation in R. GO enrichment was performed using *gprofiler2* (*gost*) (102), and GSEA was conducted using *clusterProfiler* (103), with the MSigDB Hallmark gene sets (104). RNA-Seq data have been deposited in NCBI’s Gene Expression Omnibus (GEO) with the accession number GSE315191.

### Statistics.

Data are presented as mean ± SD unless otherwise indicated in the figure legends. Statistical analyses were performed using GraphPad Prism 10. Comparisons were made using unpaired 2-tailed Student’s *t* test, 1- or 2-way ANOVA with Tukey’s post hoc correction, or χ^2^ tests, as appropriate. A *P* value of 0.05 or less was considered statistically significant. For in vivo studies, *n* = 10–22 age- and sex-matched mice were used per group, and experiments were independently repeated at least twice. Sample sizes were based on power calculations unless otherwise specified, and experimental design and matching criteria were consistent with prior studies (18, 25, 30, 31, 36–38, 61, 86–97).

### Animal study approval.

All animal housing and experimental procedures were approved by the IACUC of SUNY Upstate Medical University (protocol 443) and were conducted in accordance with institutional and federal guidelines.

## Author contributions

FM, HX, AM, MSH, SM, RT, MM, SL and TD performed experiments. LC conducted histological analysis. JMS provided CAT-Tg mice. MK designed experiments, analyzed the data, wrote the manuscript, and provided reagents.

## Funding support

This work is the result of NIH funding, in whole or in part, and is subject to the NIH Public Access Policy. Through acceptance of this federal funding, the NIH has been given a right to make the work publicly available in PubMed Central.

- SUNY Upstate Medical University Cancer Center 1184369 to MK.
- Carol Baldwin Breast Cancer Research PTA 1192750 to MK.
- NIH Institute on Aging grant 1R21AG098389 to MK.
- Paige Butterfly Run Fund grant 33875 to MK.
- Intramural Research Program of the NIH (to JMS).

## Supplementary Material

Supplemental data

Unedited blot and gel images

Supporting data values

## Version 1 01/27/2026

In-Press Preview

## Version 2 03/23/2026

Electronic publication
