# Supplementary material for: β-Catenin stabilization protects against alveolar hemorrhage through amphiregulin- and BATF-mediated Tregs
Source: JCI Insight. 2026 Jan 27;11(6):e201552. doi: 10.1172/jci.insight.201552 (PMC13043093; doi:10.1172/jci.insight.201552)
Supplement: Supplemental data [file jciinsight-11-201552-s256.pdf]

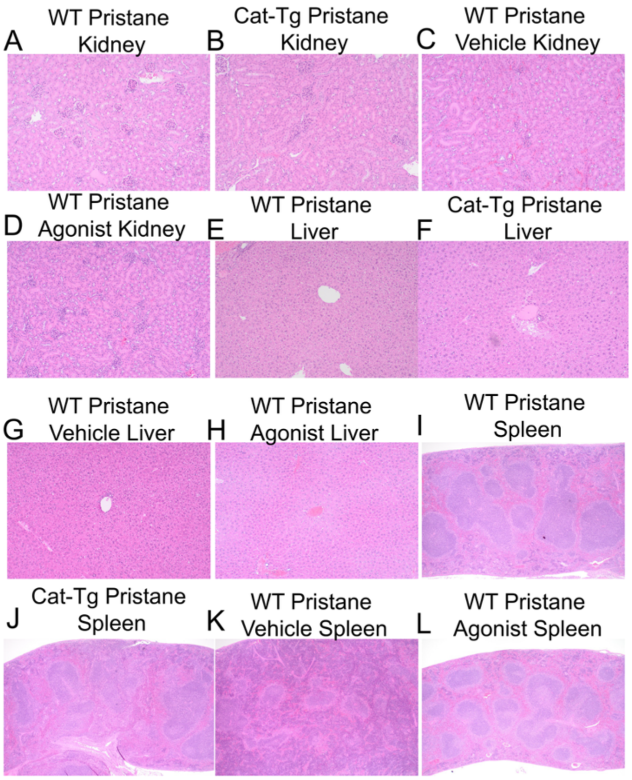

Supplemental Figure 1. Short-term pristane exposure does not induce kidney, liver, or spleen pathology. (A, B) Representative H&E-stained kidney sections from WT and CAT-Tg mice at day 14 after pristane injection, showing no detectable injury. (C, D) Representative H&E-stained kidney sections from pristane-injected WT mice treated with vehicle or β-catenin agonist, showing no detectable injury. (E, F) Representative H&E-stained liver sections from WT and CAT-Tg mice at day 14 after pristane injection, showing no detectable injury. (G, H) Representative H&E-stained liver sections from pristane-injected WT mice treated with vehicle or β-catenin agonist, showing no detectable injury. (I, J) Representative H&E-stained spleen sections from WT and CAT-Tg mice at day 14 after pristane injection, showing no detectable injury. (K, L) Representative H&E-stained spleen sections from pristane-injected WT mice treated with vehicle or β-catenin agonist, showing no detectable injury. Each experiment was repeated 3 times.

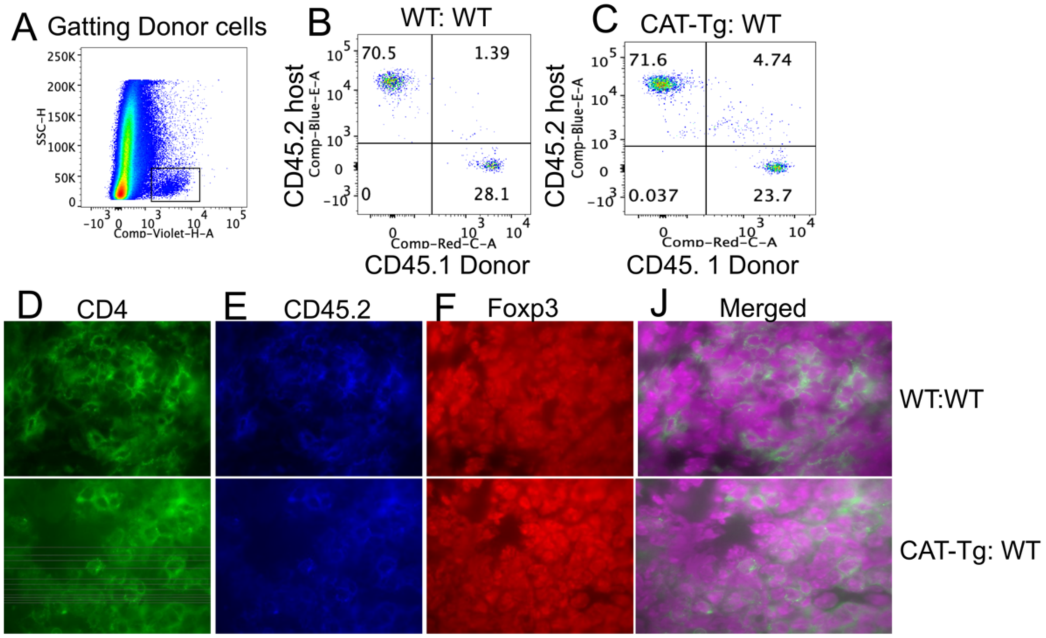

Supplemental Figure 2. Donor Tregs persist in recipient mice and traffic to the lung. (A–C) Congenic adoptive transfer strategy. Tregs isolated from WT or CAT-Tg donor mice were transferred into congenic WT recipients on a C57BL/6 background. At day 14 post-transfer, spleens were harvested and $CD3^{+}$ T cells were analyzed by flow cytometry to distinguish donor-derived versus host-derived populations based on CD45.1/CD45.2 expression, as indicated. (D–J) Immunofluorescence microscopy of lung sections confirming the presence of donor Tregs in inflamed lung tissue. Sections were stained for CD4, the donor congenic marker (CD45.1 or CD45.2, as indicated), and FOXP3, with merged images demonstrating colocalization; nuclei were counterstained with DAPI. Each experiment was repeated 3 times.

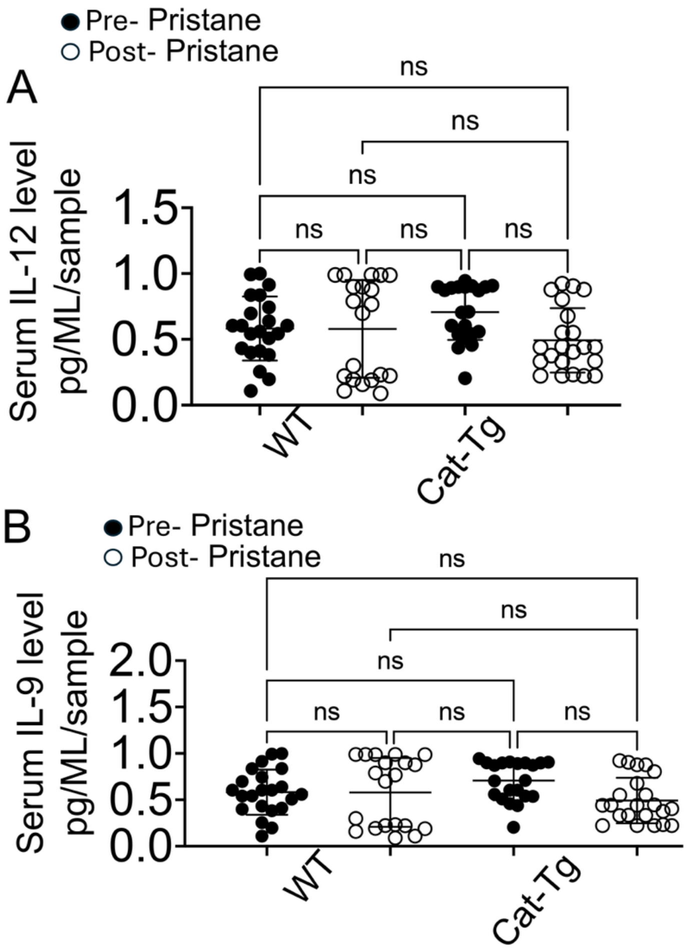

Supplemental Figure 3. β-catenin stabilization does not alter IL-12 or IL-9 during alveolar hemorrhage. (A, B) Serum IL-12 and IL-9 levels were quantified at baseline (pre-pristane) and after pristane-induced AH in WT, CAT-Tg, and β-catenin cKO mice using a multiplex bead-based immunoassay (LEGENDplex™, BioLegend). Data are presented as mean ± SEM; sample sizes (n = 15–25 mice per group) are indicated in panels. Statistical significance was determined using two-tailed Student's *t* test, one-way ANOVA, or two-way ANOVA as appropriate; NS denotes not significant. Each experiment was repeated 3 times.

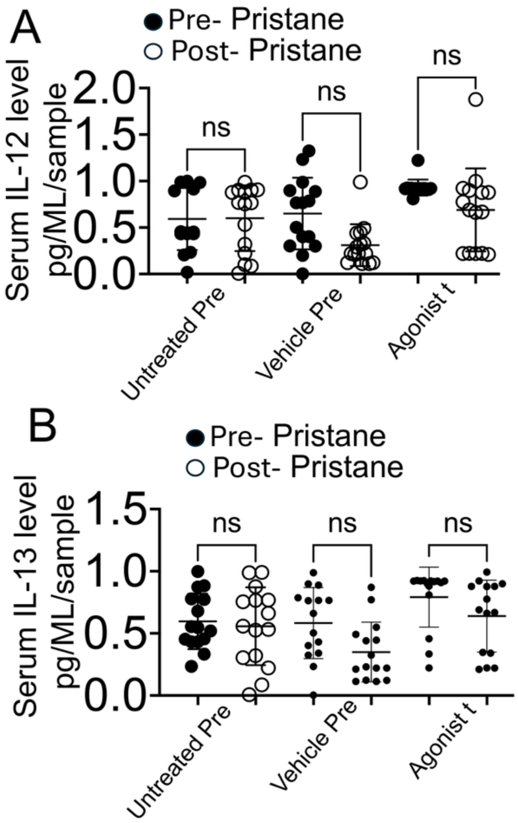

Supplemental Figure 4. β-catenin agonist treatment does not alter IL-12 or IL-13 during AH. (A, B) Serum IL-12 and IL-13 levels were quantified at baseline (pre-pristane) and after pristane-induced AH in WT mice treated with vehicle or β-catenin agonist using a multiplex bead-based immunoassay (LEGENDplex™, BioLegend). Data are presented as mean ± SEM; sample sizes (n = 15–25 mice per group) are indicated in panels. Statistical significance was determined using two-tailed Student's *t* test, one-way ANOVA, or two-way ANOVA as appropriate; NS denotes not significant. Each experiment was repeated 3 times.
