# Supplementary material for: β-Catenin stabilization protects against alveolar hemorrhage through amphiregulin- and BATF-mediated Tregs
Source: JCI Insight. 2026 Jan 27;11(6):e201552. doi: 10.1172/jci.insight.201552 (PMC13043093; doi:10.1172/jci.insight.201552)
Supplement: Unedited blot and gel images [file jciinsight-11-201552-s257.pdf]

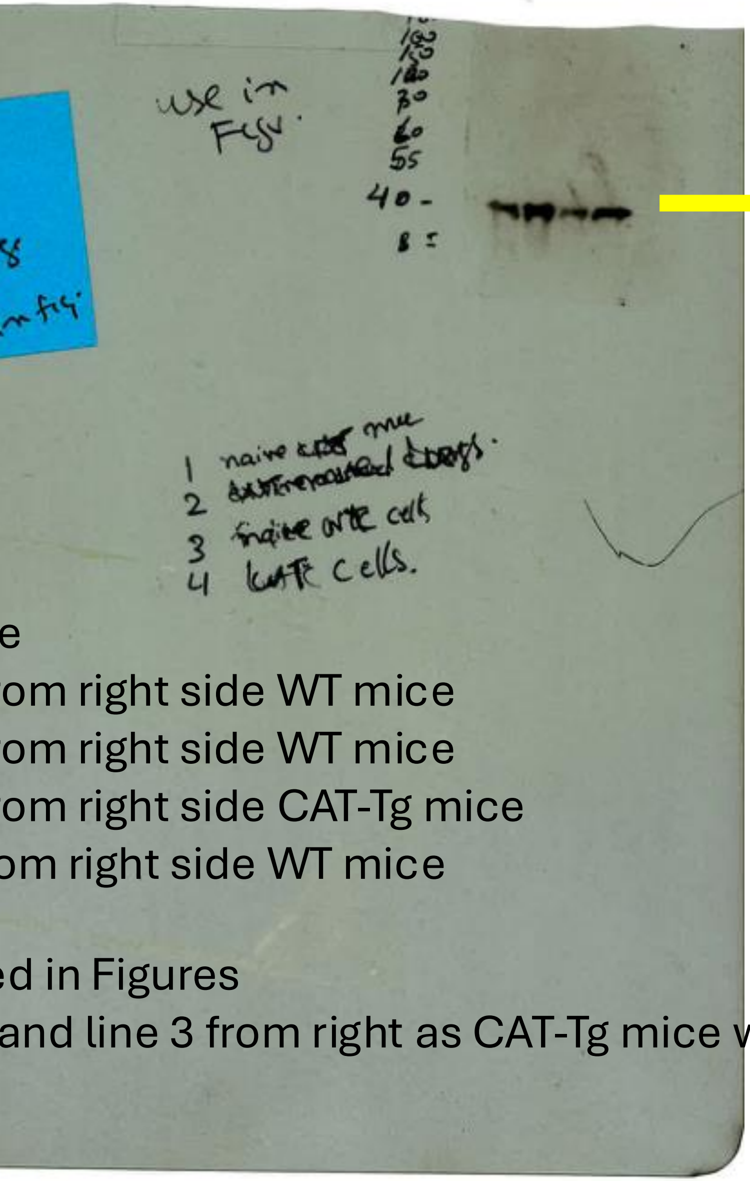

e
om right side WT mice
om right side WT mice
om right side CAT-Tg mice
om right side WT mice

ed in Figures
and line 3 from right as CAT-Tg mice were use

rotein the same gel, we run blotted for BAFT
rotein in another gel were bloated for Actin

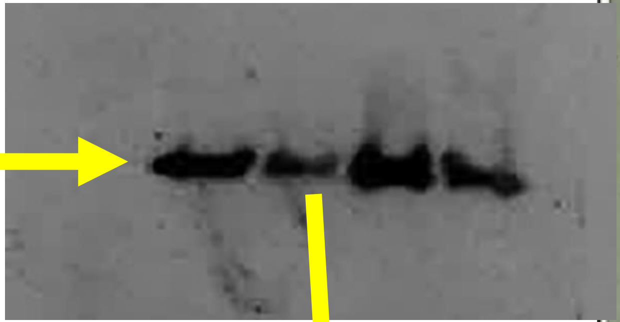

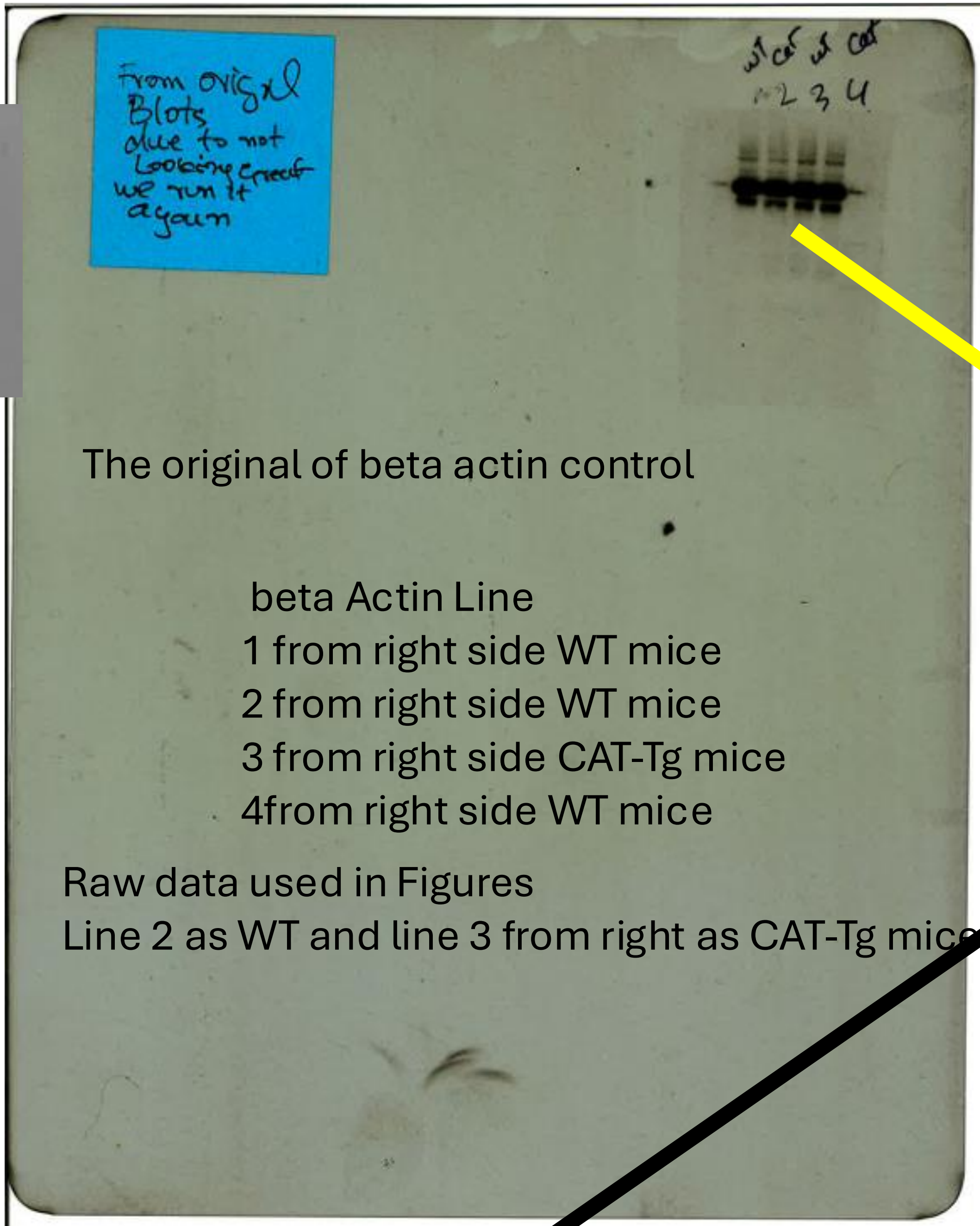

The original of beta actin control

beta Actin Line
1 from right side WT mice
2 from right side WT mice
3 from right side CAT-Tg mice
4from right side WT mice

Raw data used in Figures
Line 2 as WT and line 3 from right as CAT-Tg mice were us

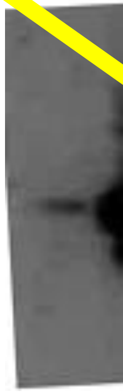

T
T-Tg

(1) Raw data

(2) Use in Photosho

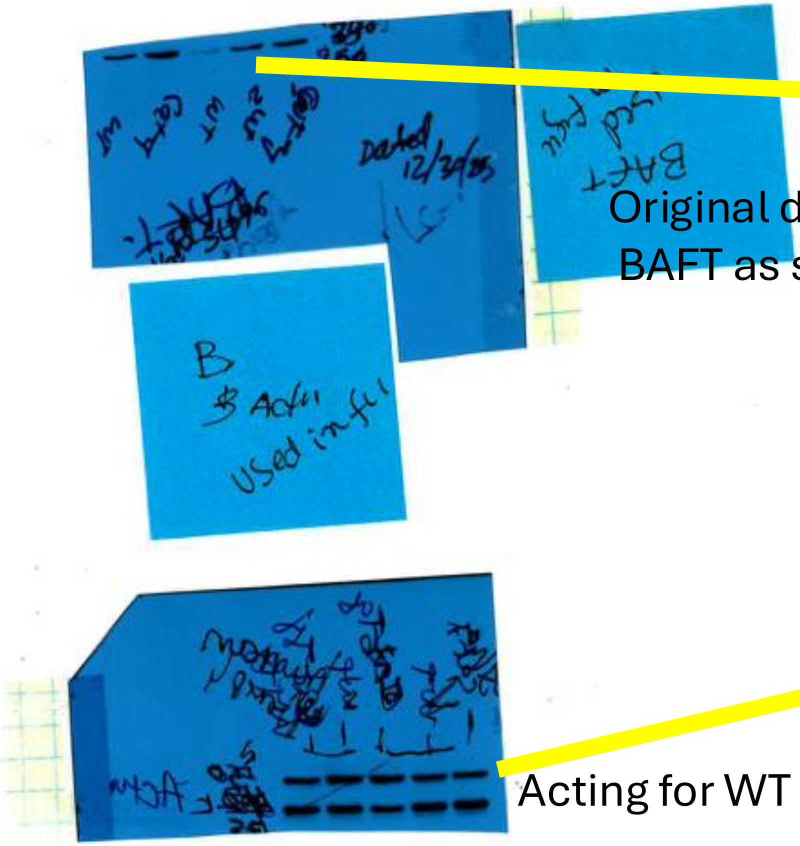

Original data use in Fig the top line CAT-Tg and WT for BAFT as shown

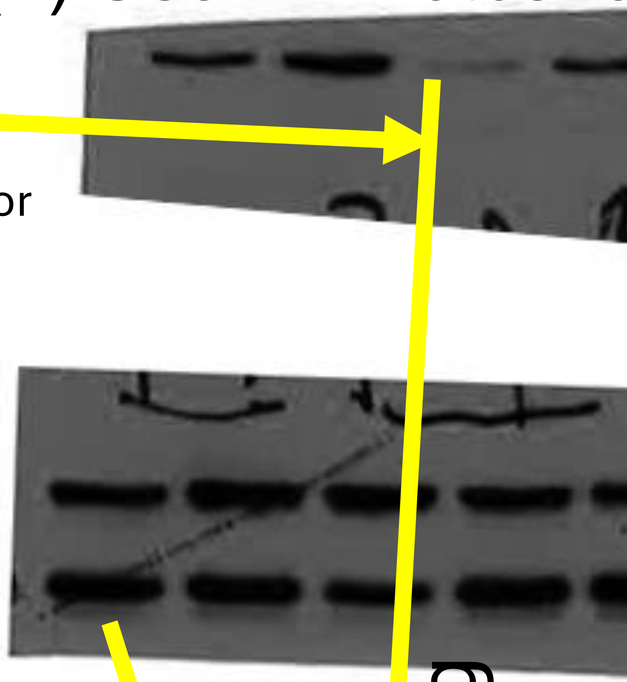

Acting for WT can Cat-Tg as used in Fig.5

The same protein the same gel, we run blotted for BAFT
The same protein in another gel were bloated for Actin

(3) Construct Figs

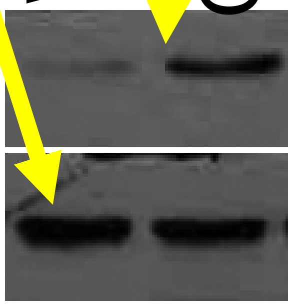

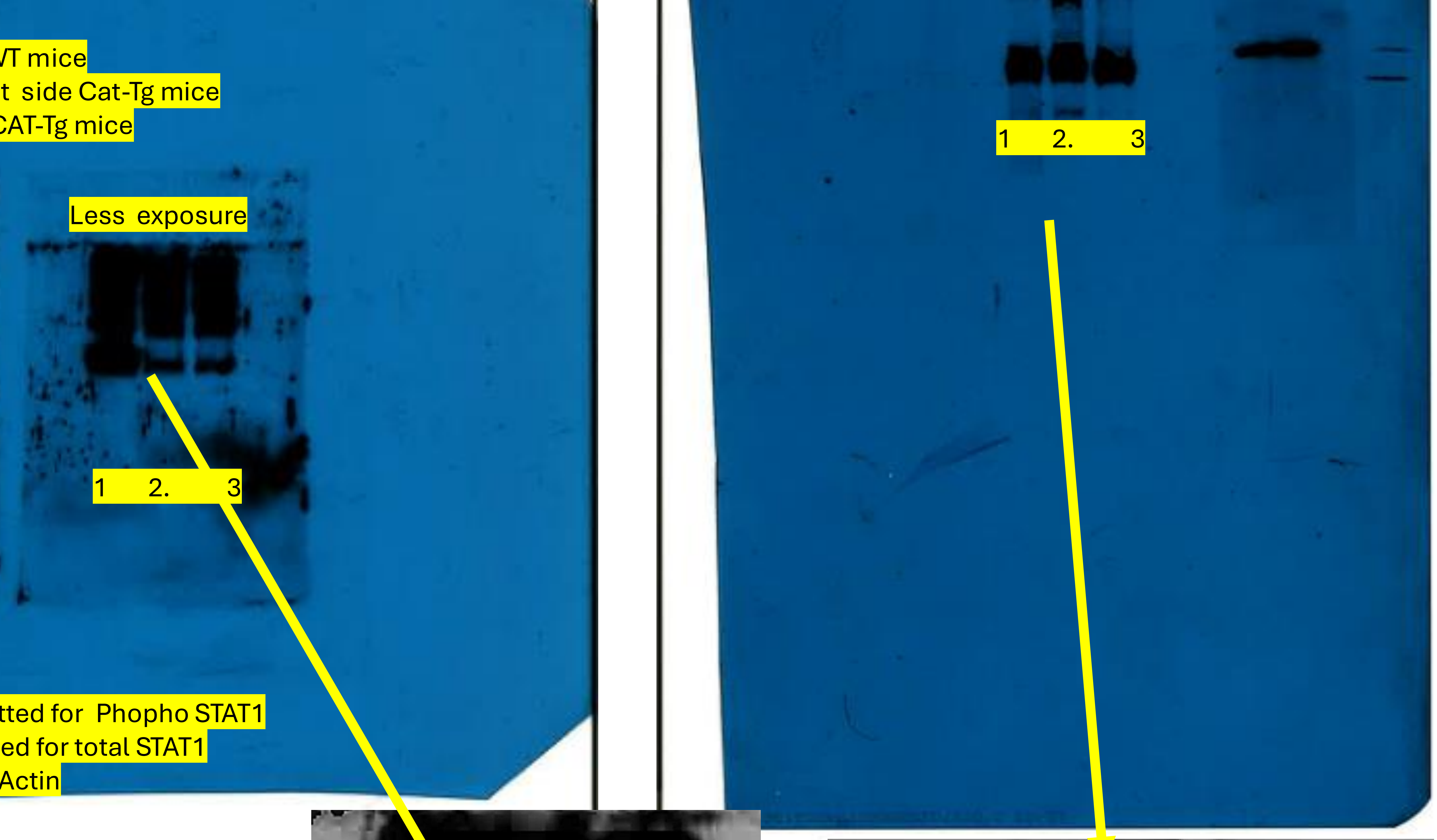
T mice
t side Cat-Tg mice
CAT-Tg mice
Less exposure
1 2. 3
1 2. 3
tted for Phopho STAT1
ed for total STAT1
Actin

STAT3

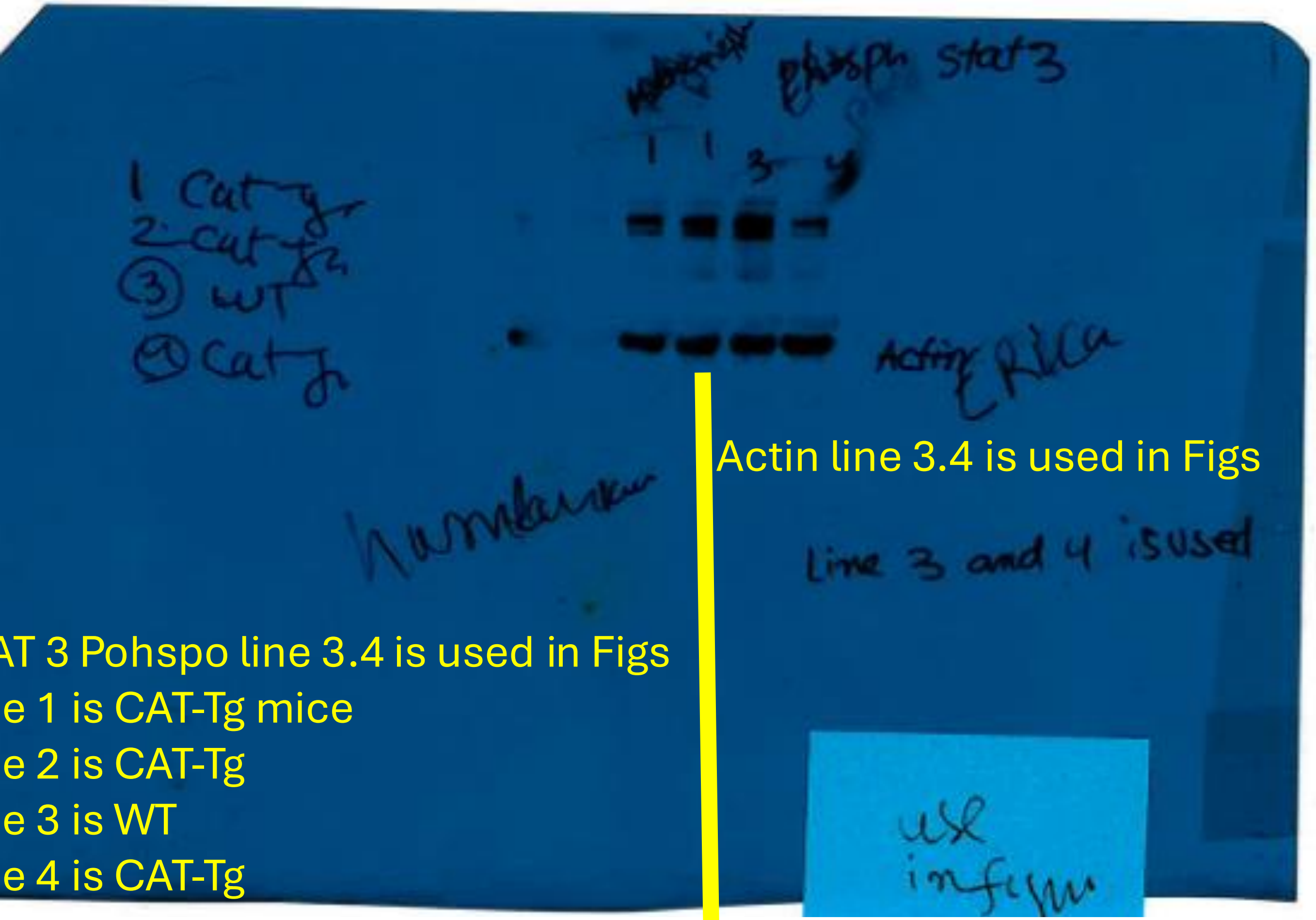

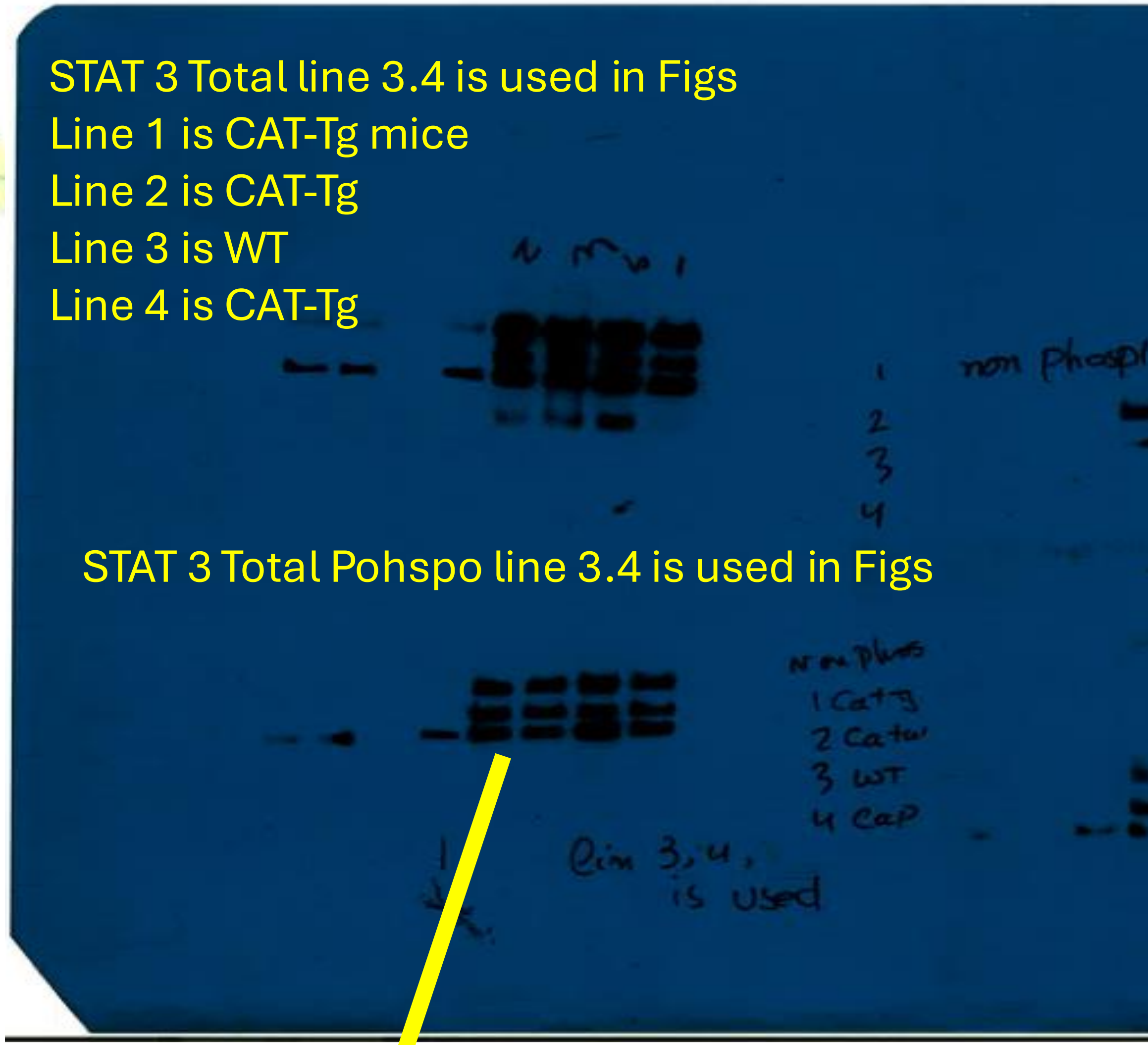

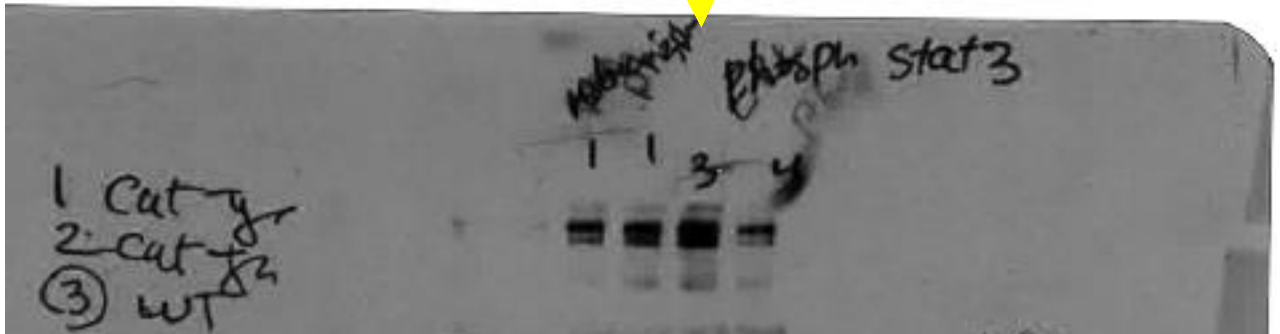

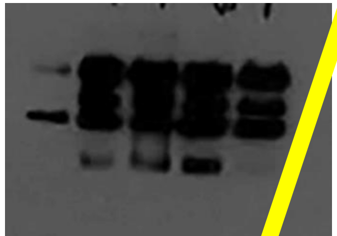

Final Figure was contrac

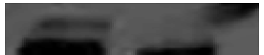

pSTAT3

pJAK

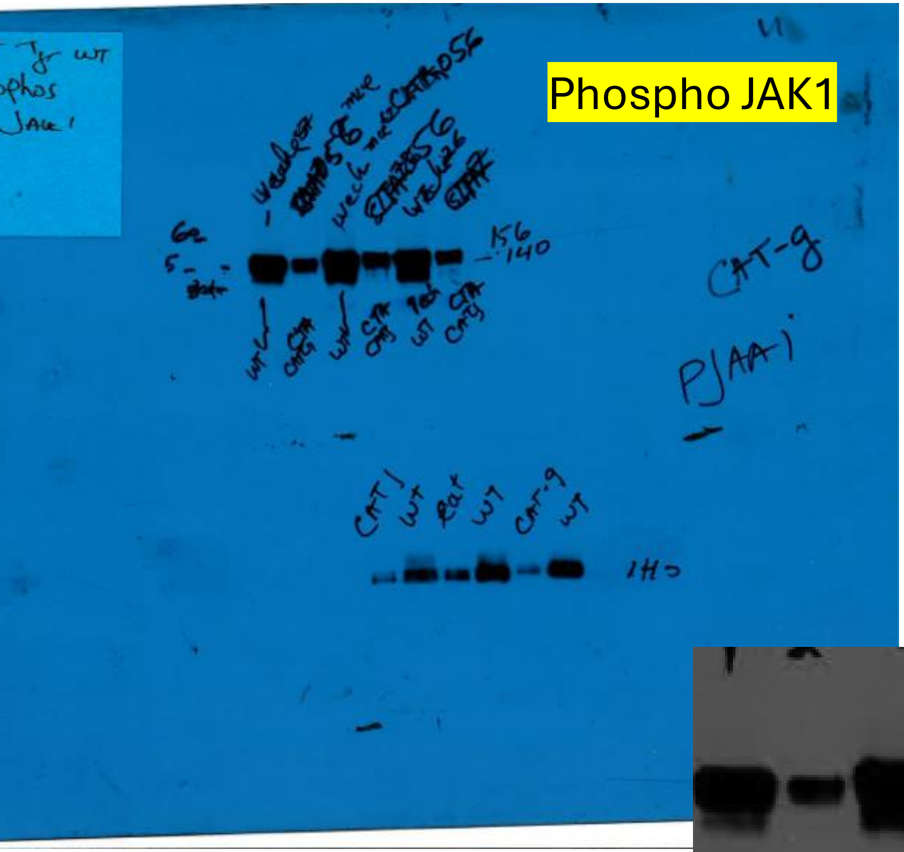

pJAK

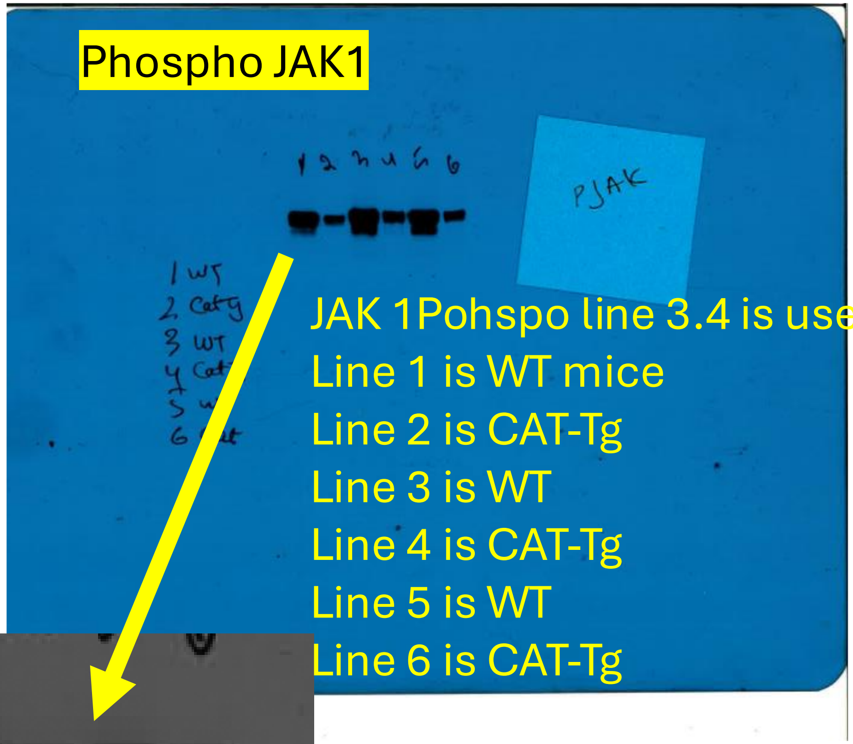

JAK 1Pohspo line 3.4 is used in Figs
Line 1 is WT mice
Line 2 is CAT-Tg
Line 3 is WT
Line 4 is CAT-Tg
Line 5 is WT
Line 6 is CAT-Tg

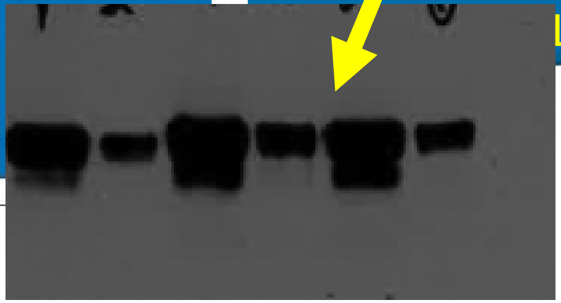

Total JAK

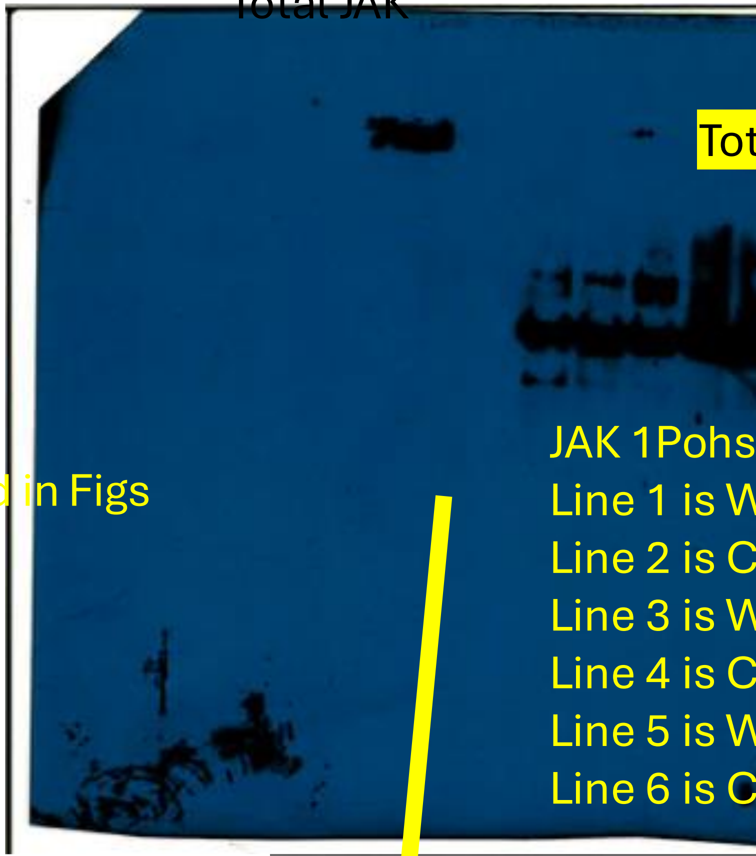

JAK 1Pohs
Line 1 is W
Line 2 is C
Line 3 is W
Line 4 is C
Line 5 is W
Line 6 is C

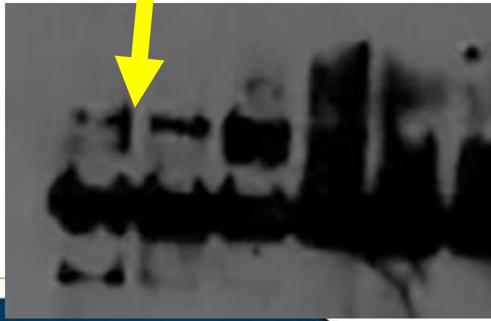

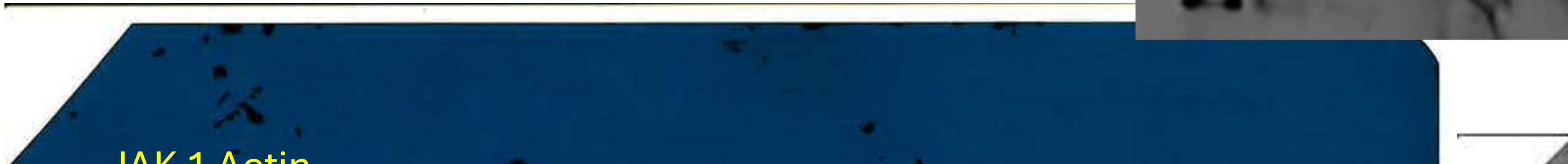

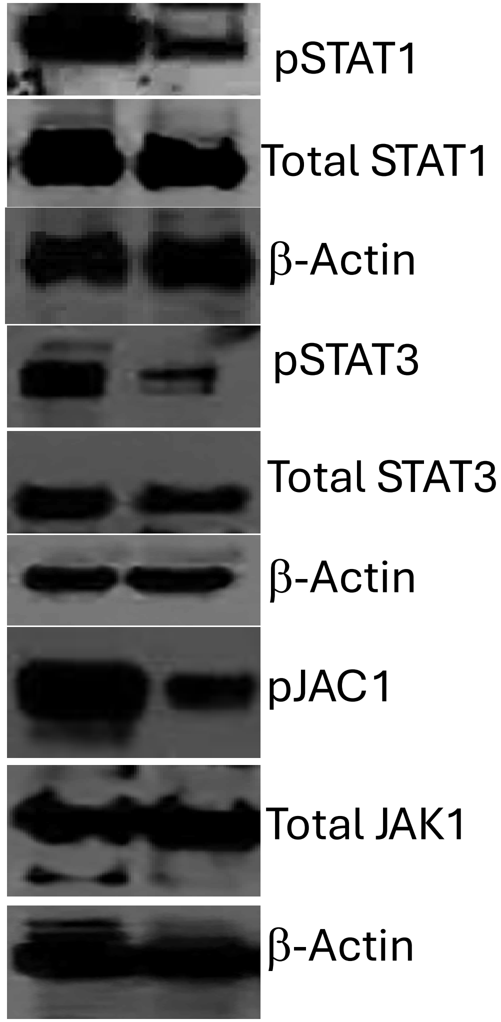

pSTAT1
Total STAT1
β-Actin
pSTAT3
Total STAT3
β-Actin
pJAC1
Total JAK1
β-Actin
